# Magnetic Resonance Imaging Techniques for Brown Adipose Tissue Detection

**DOI:** 10.3389/fendo.2020.00421

**Published:** 2020-08-07

**Authors:** Mingming Wu, Daniela Junker, Rosa Tamara Branca, Dimitrios C. Karampinos

**Affiliations:** ^1^Department of Diagnostic and Interventional Radiology, School of Medicine, Technical University of Munich, Munich, Germany; ^2^Department of Physics and Astronomy, University of North Carolina at Chapel Hill, Chapel Hill, NC, United States

**Keywords:** brown adipose tissue (BAT), white adipose tissue (WAT), magnetic resonance imaging, magnetic resonance spectroscopy, morphology, activation, thermogenesis

## Abstract

Magnetic resonance imaging (MRI) and magnetic resonance spectroscopy (MRS) methods can non-invasively assess brown adipose tissue (BAT) structure and function. Recently, MRI and MRS have been proposed as a means to differentiate BAT from white adipose tissue (WAT) and to extract morphological and functional information on BAT inaccessible by other means. Specifically, proton MR (^1^H) techniques, such as proton density fat fraction mapping, diffusion imaging, and intermolecular multiple quantum coherence imaging, have been employed to access BAT microstructure; MR thermometry, relaxometry, and MRI and MRS with ^31^P, ^2^H, ^13^C, and ^129^Xe have shown to provide complementary information on BAT function. The purpose of the present review is to provide a comprehensive overview of MR imaging and spectroscopy techniques used to detect BAT in rodents and in humans. The present work discusses common challenges of current methods and provides an outlook on possible future directions of using MRI and MRS in BAT studies.

## Introduction

### Role of Biomedical Imaging in BAT Research

Even though the presence of brown adipose tissue (BAT) in human adults had been reported back in 1972 (1), its relevance in adult humans has largely been neglected by the scientific and medical community until 2009.

*In vivo* detection of BAT activity in fluorine-18 fluorodeoxyglucose (^18^F-FDG) positron emission tomography (PET) scans dates back to 1996 (2). However, only in 2002 co-registered PET and computed tomography (CT) images allowed to recognize that the increased symmetrical ^18^F-FDG uptake seen in the cervical and thoracic spine region of cancer patients, initially attributed to muscular uptake, was due to activated BAT (3). Initially seen as nuisance, the detection of active BAT in adult humans sparked a scientific curiosity only after 2009, when three simultaneous publications in the *New England Journal of Medicine* (4–6) reported that the glucose-avid adipose tissue detected in adult humans on PET/CT scans had been confirmed to be BAT by adipose tissue biopsies and molecular biology analyses (4–6).

In the last 10 years, a variety of imaging techniques have been deployed to detect and understand the role of BAT in humans. While for ethical reasons biopsies of human BAT have been limited to mostly cadavers or tissues excised as part of oncological procedures (7), biomedical imaging techniques have enabled us to study the morphology and function of this tissue *in vivo*. Tomographic imaging techniques such as PET, CT, and magnetic resonance imaging (MRI) have enabled the detection of BAT in both rodents and humans, while providing spatially resolved information on its morphology and functional properties. The large amount of ongoing studies and recent publications involving biomedical imaging of BAT speaks both for the vast interest in BAT research and for the importance of high-quality non-invasive imaging.

The present review introduces briefly the main imaging modalities used to research BAT in rodents and humans. Then, it focuses on current MR methods that have shown to provide both morphological and functional information on BAT. The underlying properties and limitations of MR techniques applied in BAT research are also discussed in a comprehensive way. Finally, an outlook on possible future directions is provided.

### Biomedical Imaging Modalities for BAT Research

An overview of the different imaging modalities in BAT research is given in Table 1.

**Table 1:** Comparison of the different imaging modalities (including strengths and weaknesses).

	**Free of** **ionizing radiation**	**Free of injections**	**Independence on current BAT- activity**	**Quantitative imaging possible**	**Spatial resolution**	**3D possible**	**Penetration depth**	**Required scan time**	**Costs for hardware**
PET^ 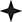 ^	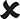	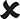	( 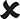 , ✓) radiotracer dependent	(✓)	Low >1 mm^3^	✓	Unlimited	Long	High
CT^ 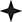 ^	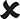	✓	✓ HU is hydration- dependent	✓	Medium <1 mm^3^	✓	Unlimited	Short (seconds)	Medium
CEUS^ 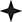 ^	✓	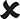	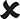	(✓)	Medium <1 mm^3^	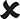	Limited	Short	Low (~10.000 USD)
Optical techniques	✓	( 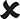 )	( 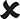 )	✓	High <<1 mm^3^	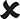	Low	Short	Low
MRI^ 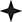 ^	✓	(✓, 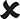 )	✓	✓	Medium ~1 mm^3^	✓	Unlimited	Long (minutes)	High (~1 Mio USD per Tesla)

*Techniques that the co-authors have experience with are marked with a^
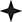
^. 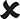 means no and ✓ stands for yes. The parentheses indicate that the statement is true only under certain circumstances*.

Since 2009, most BAT imaging studies have used ^18^F-FDG-PET to identify activated BAT in humans (8), despite its many known limitations (9): First, the exposure to ionizing radiation is a major concern when applying PET for studying BAT, especially for longitudinal studies in healthy, young, or pediatric populations. Second, fatty acids hydrolyzed from intracellular triglycerides, not glucose, are the main substrates for BAT oxidative metabolism (10). Because in BAT, glucose is used mainly for *de novo* lipogenesis, which is highly dependent on intracellular lipid content, glucose uptake in BAT may not always reflect its thermogenic activity (11). Therefore, the underlying assumption that glucose uptake reflects the thermogenic activity of the tissue has recently been questioned (12, 13). While fatty acid tracers would be a better probe of BAT thermogenesis, competition between exogenous fatty acid tracers and intracellular fatty acids released from intracellular triglycerides practically leads to little contrast between BAT and white adipose tissue (WAT) (14). Finally, while some radiotracers may not rely on BAT activation (15) for BAT detection, the most commonly used radiotracer, ^18^F-FDG, does rely on BAT activation. Specifically, inactive BAT will not be identified in ^18^F-FDG PET scans, while different degrees of BAT activation, coupled with the poor test–retest reliability of FDG PET/CT in measuring BAT glucose metabolism (16), is expected to lead to a great variation in estimates of BAT volumes, from as low as 10 ml (17) to as much as 650 ml (18).

Because of its low anatomical spatial resolution, PET is usually combined with either CT (PET/CT) or MRI (PET/MRI). In PET/CT, metabolically active BAT is identified as a tissue with a radiodensity between −100 and −30 Hounsfield units (HU) on the CT images (19). During activation, a further increase in tissue radiodensity of about 10 HU can be observed in supraclavicular regions known to contain BAT (20). However, due to the broad overlap of HU values between BAT and WAT, it remains nearly impossible to differentiate BAT from WAT solely based on a single CT measurement. Dual-energy CT (DECT) offers the possibility to measure tissue attenuation at two different energies, and it has been suggested as a way to differentiate BAT from the less hydrated WAT (21). However, radiation exposure associated with CT examinations makes longitudinal CT examinations in healthy volunteers unfeasible.

Small studies in mice and humans have also investigated the use of contrast-enhanced ultrasound (CEUS) for BAT detection (22, 23). During adrenergic stimulation in mice and cold activation in humans, an increase in BAT blood flow was detected by CEUS. The low cost of US is attractive for the investigation of BAT activation, especially when searching for BAT-targeted therapies. However, in obese subjects, tissue hypertrophy leads to a reduction in tissue vascular density as well as to a reduction in tissue blood flow (24). Since BAT detection and differentiation from WAT with CEUS is based on the higher vascular density of BAT and on the increase in tissue blood flow during stimulation, it is expected to be less effective in animal models of obesity or in obese subjects.

Optical techniques are an emerging tool for BAT imaging, especially in rodents. It has been shown that Cerenkov luminescence imaging (CLI) with ^18^F-FDG can be used to optically image BAT in small animals (25). Even though cheaper and faster than PET, CLI again comes with the need for radiotracer injection. Near-infrared time-resolved spectroscopy (NIR_TRS_) has also been used in humans to provide separate independent information on tissue absorption and scattering of BAT. Specifically, a significant correlation was found between ^18^F-FDG-PET standardized uptake values (SUV) of BAT activity and tissue scattering properties in the supraclavicular region, potentially related to oxy- and deoxy-hemoglobin concentration in the tissue (26). However, these measurements reflect tissue distribution and content in the entire region of interest, including skin, muscle, and subcutaneous WAT that can be present in the region, making the interpretation of the results a challenge. NIR fluorescence imaging allows quantitation of BAT perfusion and thus provides an indirect measurement of BAT activity. The requirement of a fluorescent dye, however, precludes its use in humans (27). Real-time sensing of hemoglobin by multispectral optoacoustic tomography (MSOT) was recently described as a label-free non-invasive imaging tool of BAT activation that enables longitudinal measurements in individual subjects (28). However, MSOT only renders cross-sectional images. Thus, volumetric quantification of BAT mass can become challenging. Furthermore, by using light as a probe, MSOT as any other optical imaging technique has a limited penetration depth (2–5 cm), which can be a challenge for its adoption in humans or for the detection of BAT in overweight and obese individuals.

### MRI for BAT Research

MRI provides superior tissue contrast and adequate spatial resolution when compared to other tomographic imaging techniques. Contrast in MR images is determined by the chemical composition and microstructure of the tissue of interest, which directly affect frequency, density, diffusion, and relaxation properties of the detected nuclear spins. By careful design of the MR pulse sequence, one property can be emphasized over another. Therefore, one could imagine to leverage on the MR-detectable endogenous differences between WAT and BAT to differentiate these two tissues. By virtue of being radiation-free, MR can be safely used in longitudinal studies in human subjects of all age groups, aiming at understanding how BAT evolves over the course of a lifetime.

MR methodologies for studying BAT and applications of MRI techniques for probing BAT in health and disease have been previously summarized in publications reviewing the use of different imaging modalities in BAT research (9, 21, 29–36). Some of the MRI techniques used to detect BAT have also been reviewed as part of techniques used for the non-invasive assessment of different fat and adipose tissue depots (37, 38). However, only two review papers have focused entirely on MRI techniques used to detect BAT in the research setting (39, 40). The present work aims at a more comprehensive and updated review of the state-of-the-art MR techniques and applications for BAT research.

An electronic search in PubMed (http://www.ncbi.nlm.nih.gov/pubmed) was performed without a starting date up to February 2020 using as search terms “brown adipose tissue” and one of the following terms: “Magnetic Resonance Imaging” or “Magnetic Resonance Spectroscopy.” The search results included investigations in rodents and/or in humans. The reference lists of relevant articles were also screened.

The following sections include a critical assessment of current MR techniques used to detect BAT, most of which have been used by the co-authors of the present review.

### Overview of MR Contrast Mechanisms for BAT Detection

**Figure 1:**
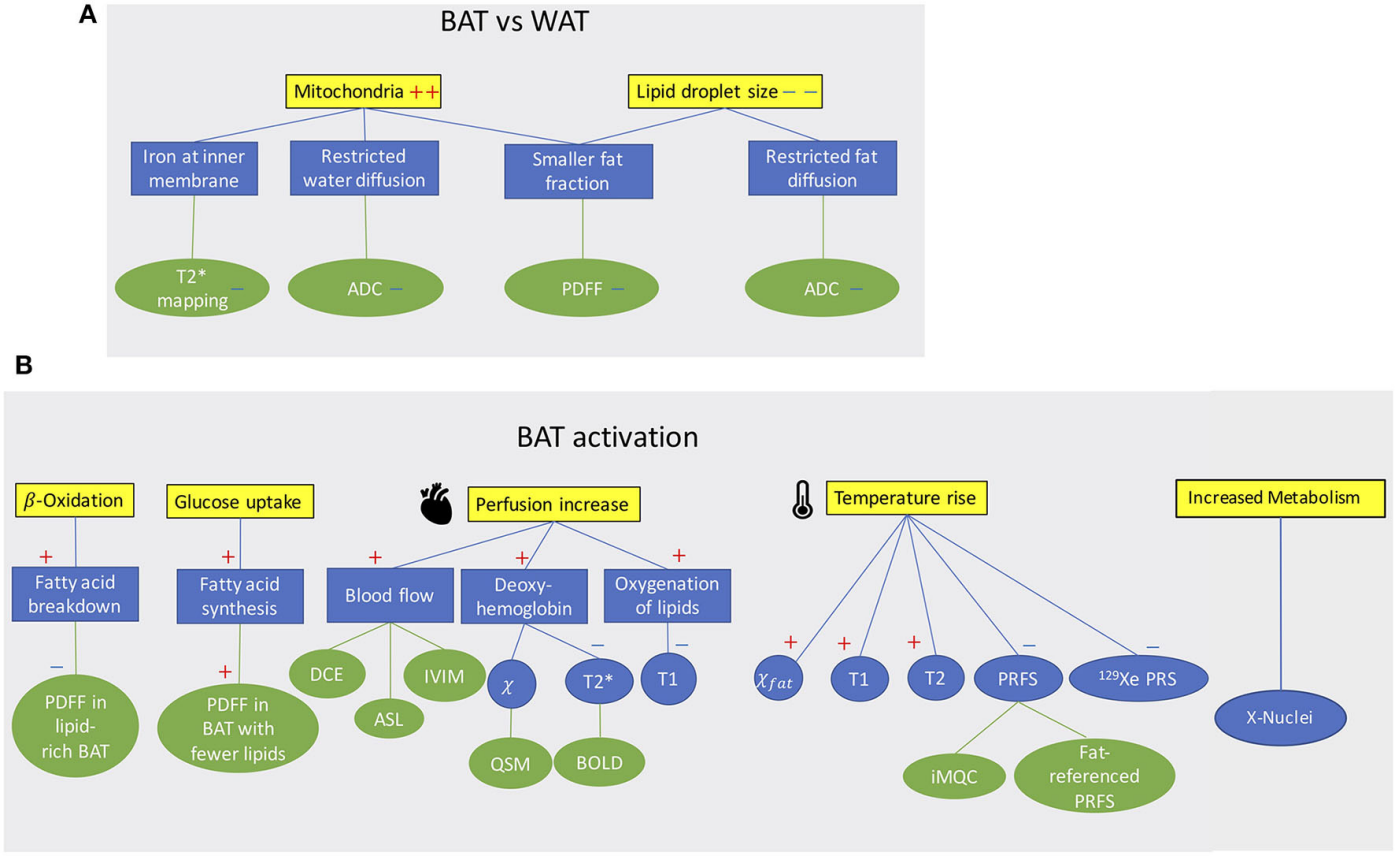
**(A)** Conceptual schematic highlighting morphological differences between BAT and WAT detectable with MR contrast. **(B)** Conceptual schematic of MR contrast mechanisms for detecting BAT function. A “+” sign indicates an increase a “–” sign indicates a decrease.

**Figure 2:**
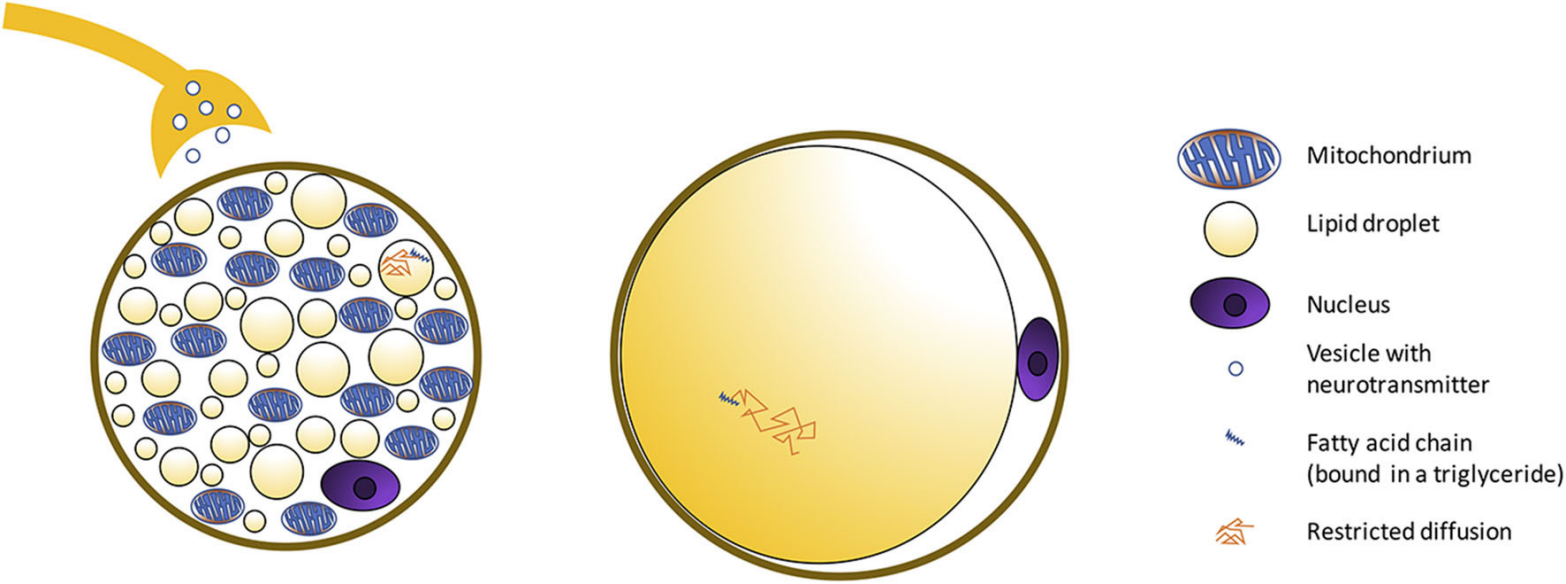
Schematic showing morphological differences between BAT and WAT cells. BAT is composed of adipocytes much smaller in size than white adipocytes, and with a cytoplasm occupied by multiple small lipid droplets (multilocular adipocytes), often of less than few micrometers in diameter, distributed uniformly throughout the entire depot. As a result, compared to classical white adipose tissue, where a single fat droplet occupies most of the cell cytoplasm, brown adipocytes appear much more hydrated, with a water content typically ranging between 50 and 70%. The presence of numerous mitochondria inside the BAT cell with UCP1 in the mitochondrial membrane (Figure 3) allows for its vivid metabolism.

**Figure 3:**
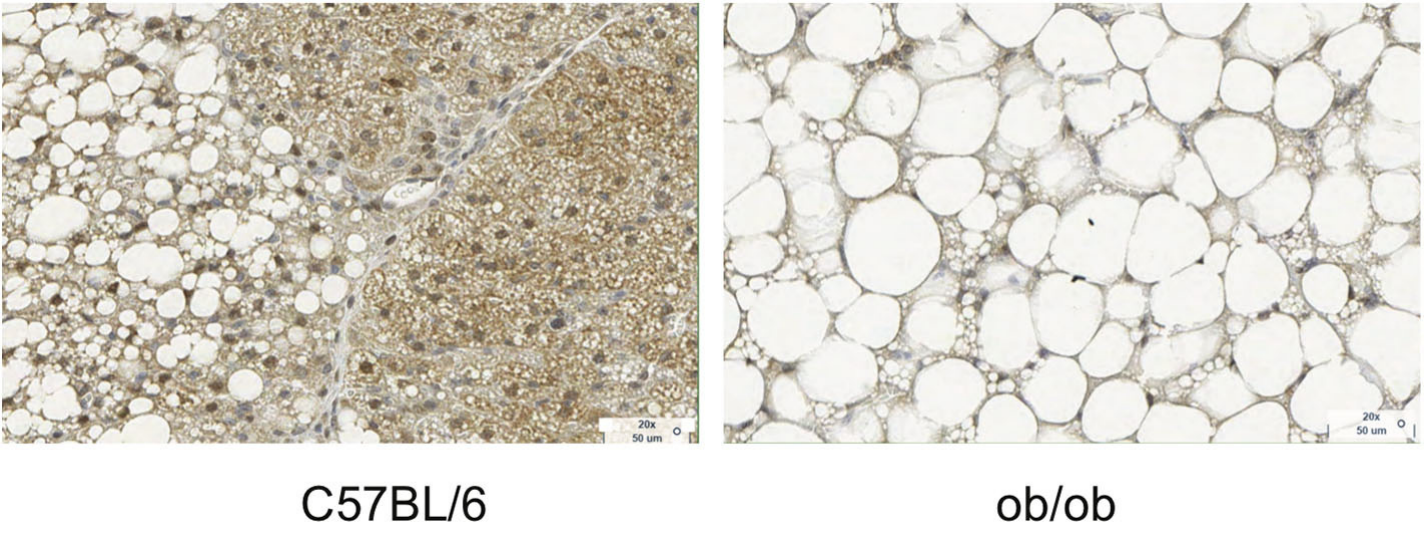
Histology images with UCP1 staining for C57BL/6 and obese phenotype mouse. This figure is original and based on data from (41).

MR can differentiate BAT from WAT based on morphological (Figure 1A) and functional (Figure 1B) differences between these two tissues. Differentiation based on morphological differences is advantageous because it does not require stimulation and activation of the tissue. Morphologically, the presence of multiple, smaller lipid droplets in BAT adipocytes makes this tissue more hydrated than WAT. Differences in tissue hydration can be measured by fat fraction mapping techniques. Furthermore, BAT is highly vascularized and brown adipocytes have numerous iron-rich mitochondria (Figures 2, 3), which are responsible for the brown color of the tissue under natural light. Both the presence of iron at the inner mitochondria membrane and magnetic susceptibility gradients generated at the many water–fat interfaces lead to a rapid signal dephasing. This effect is clearly visible in T_2_^*^-weighted as well as T_2_-weighted images, in which BAT appears darker than WAT. Microstructural differences between white and brown adipocytes translate in differences in diffusion properties of both water and fat spins in these two tissues. Because the inner mitochondrial membrane is not permeable to water, water diffusion inside and outside the mitochondria wall is greatly restricted. Restricted water diffusion is reflected in a smaller apparent diffusion coefficient (ADC) that can be measured by diffusion-weighted MRI. Similarly, because lipids are confined in smaller lipid droplets in BAT, diffusion of lipid molecules in BAT is also more restricted. A smaller fat ADC can be measured in BAT compared to WAT by diffusion-weighted MRI/MRS.

BAT activation leads to a change of many MR tissue properties (Figure 1B). After cold exposure or food intake, thermogenic activity in BAT is mediated by the sympathetic nervous system. Metabolic activity propels hydrolysis of local triglycerides and fatty acid consumption via β-oxidation. Depletion of triglycerides in BAT can be detected as a decrease in tissue fat content. At the same time, increased glucose uptake during BAT activation (glucose is expected to be used for de novo lipogenesis as well as to sustain the cell's ATP demand) and the metabolic fate of glucose could be traced by using HP ^13^C-pyruvate or ^2^H-glucose MRI.

The increased oxygen demand in BAT is met by an increase in tissue perfusion. Changes in BAT perfusion and oxygenation can be detected by several MR techniques, such as T2^*^-weighted techniques, dynamic contrast enhanced MRI, dynamic susceptibility contrast ^1^H MRI, and HP ^129^Xe MRI. Since heat production is the main function of BAT, an increase in BAT temperature can be detected and quantified by measuring temperature-sensitive MR parameters such as the water resonance frequency, T_1_ and T_2_ relaxation times of ^1^H, as well as the frequency of other temperature-sensitive nuclei such as ^129^Xe.

In the following description, MR techniques are described using the following three labels: “widely used techniques” for techniques used in multiple published studies, and “technique of limited use” indicating a method that has not become established because of certain limitations.

### Considerations for Application of Quantitative MR Techniques to BAT

Most of the previously mentioned ^1^H-based MR techniques (fat fraction, T_1_, T_2_, T_2_^*^, and diffusion-weighted) primarily measure properties of water components in tissue. However, the same techniques can be used to probe fat components. In humans and in rodents, BAT contains both water and fat spin components, something that needs to be considered when applying quantitative MRI in BAT-containing depots. Specifically, quantitative imaging techniques that take into account the presence of both water and spin components need to be adopted. Possible approaches include either the suppression of the unwanted tissue component (e.g., fat suppression) or modeling the presence of both water and fat components. As an example, BAT water and fat components are characterized by different T_1_ and T_2_ relaxation times and diffusion properties: the water component in BAT has typically shorter T_2_, longer T_1_, and a lower diffusion constant than the corresponding fat component.

## Anatomy of Bat Depots in Rodents and Humans

### Adipocyte Types

Both in rodents and in humans, different types of adipocytes can be found: white, classical brown, and beige (brown-like-in-white, brite, inducible brown) adipocytes, with the latter having an intermediate morphology between white and brown adipocytes. Brown adipocytes share a common origin with skeletal muscle cells, while white adipocytes have distinct progenitor cells (42, 43). Beige adipocytes can transdifferentiate from white adipocytes and differentiate *de novo* from progenitor cells (44). To date, different lineage markers have been found for beige adipocytes, supporting the idea that beige adipocytes might exist in multiple subtypes, depending on external stimuli (45).

Morphologically, white adipocytes exhibit a single large intracellular lipid droplet and only few mitochondria, whereas brown adipocytes have multiple small cytoplasmic lipid droplets, often of less than few micrometers in diameter, and numerous mitochondria (Figure 3). Beige adipocytes are multilocular, like brown adipocytes, but are much bigger in size. Regarding functionality, white adipocytes store excess lipids in the form of triglycerides, while brown adipocytes can either store energy in the form of fat or dissipate energy as heat. This is accomplished thanks to a protein called the uncoupling protein-1 (UCP-1), which is present in the mitochondria of brown adipocytes at high concentration and is considered a marker of brown adipocytes. Beige adipocytes are also capable of storing fat or producing heat but, compared to classical brown adipocytes, have a remarkably reduced thermogenic potential. S100 calcium-binding protein B and leptin, adipokines relevant in the regulation of physiological functions, can be found on white and beige adipocytes, but not in brown adipocytes (44).

### Rodents

**Figure 4:**
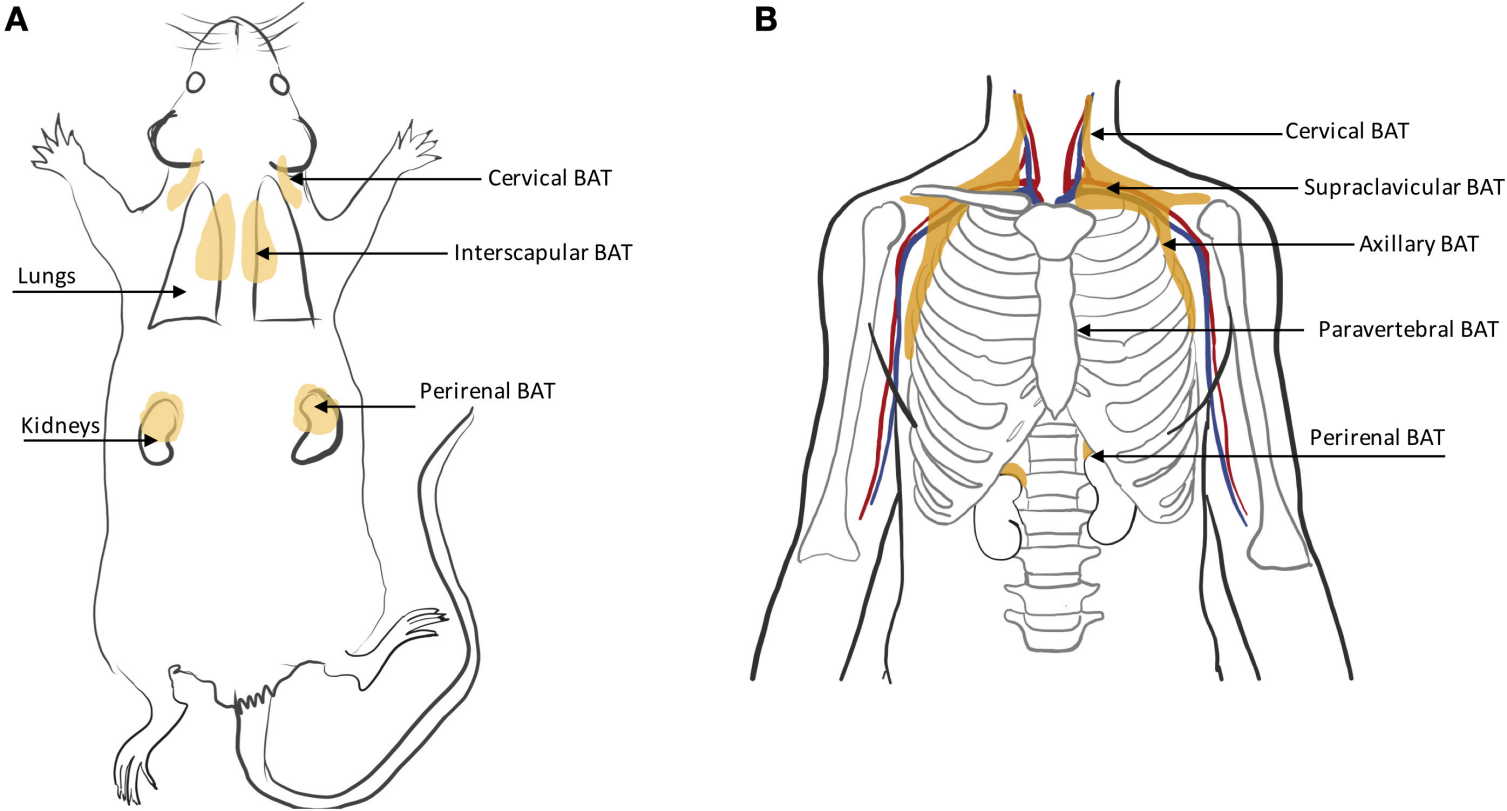
Schematic displaying major BAT depots in **(A)** rodent and **(B)** human anatomy.

In rodents, BAT is found primarily in the interscapular region and axillae, with the interscapular BAT depot (iBAT) being the largest one (11, 46) (Figure 4A). Other small depots, often only few millimeters in size, but still visible upon dissection, are often found in the supraclavicular, cervical, supraspinal, and perirenal areas. Because of their smaller size, these depots are harder to identify and differentiate from WAT. Other BAT depots are also found, upon dissection, in the anterior subcutaneous and suprascapular region, as well as in the inguinal region. These depots contain unilocular brown adipocytes with morphology and function that more closely resemble that of classical white adipocytes.

### Humans

BAT in humans is usually present in mixed depots of white, brite, and classical brown adipocytes (47, 48). Human BAT is predominantly found in the cervical, supraclavicular, axillary, mediastinal, paravertebral, and perirenal (49) regions (Figure 4B). Exposure to cold can lead to an increase in BAT volume of activity as detected by FDG-PET (50).

Due to great cellular heterogeneity of supraclavicular fat, efforts have been made to find cell-surface markers to characterize different types of cells in the region. Recently, different cell-surface markers have been identified to distinguish between brown, beige, and white adipocytes in human biopsies (51, 52). For example, BAT in human infants appears similar to classical rodent BAT (48), while beige marker proteins can be found as well (53). Since no imaging methods are capable of differentiating classical brown adipocytes from beige adipocytes yet, the described mixture of different cell types will hereinafter be referred to as “BAT.”

## Characterizing Bat Water–Fat Composition

The MR protocol most commonly used to assess BAT morphology and to differentiate BAT from WAT is based on the quantification of water and fat content in the tissue. Because, compared to WAT, BAT has a reduced fat content (Figures 2, 3), quantification of water–fat composition in tissues can be used to differentiate BAT from WAT. Single-voxel proton MRS or chemical shift encoding-based water–fat MRI are the two main approaches used to quantify water–fat composition in tissues with MR (54).

### Single-Voxel Proton MR Spectroscopy [Technique of Limited Use]

Single-voxel proton MRS can be used to quantify water and fat content within a small 3D volume. MRS can spectrally resolve the different fat peaks and thus measure adipose tissue fatty acid composition (55). If single-voxel MRS is performed with variable T_1_- and T_2_-weightings, it can also be used to extract relaxation properties of water and fat components in BAT (55). However, when applied to the heterogeneous fat tissue in the human supraclavicular fossa, single-voxel MRS measurements can be challenging. Breathing motion and magnetic susceptibility gradients between water and fat compartments significantly broaden the spectral lines, making it difficult to correctly quantify water and fat spin components. A reduction of the voxel size rarely helps, as magnetic susceptibility gradients between water and fat compartments are quite strong, both at a macroscopic and microscopic level. In addition, breathing motion further complicates shimming procedures and leads to voxel mis-registration across averages, which can become particularly severe when small voxels are employed. Finally, to extract the proton density fat fraction (PDFF), MRS acquisitions need to employ a long repetition time (TR), to minimize T_1_ weighting effects, and an acquisition with multiple echo times (TE) to correct for T_2_ weighting effects. Long acquisition times, coupled with voxel mis-registration due to physiological motion, cause severe spectral line broadening, hampering correct quantification of water and fat spin components in the region.

### Chemical-Shift-Encoded MRI [Widely Used Technique]

While early MR-based BAT fat fraction measurements were based on frequency selective excitation methods to collect a signal-weighted fat fraction image (56, 57), recently chemical shift encoding methods have become widely used for BAT detection. These methods are able to quantify the PDFF, defined as the proportion of mobile proton density in tissue attributable to fat, which is nowadays considered a standardized imaging biomarker of tissue fat content (58). PDFF has shown excellent linearity, accuracy, and precision across different field strengths and scanner manufacturers for liver applications (59) and is thus used to assess fat infiltration in liver, abdominal organs (54), and bone marrow (60). Chemical shift encoding-based water–fat imaging combines a (2D or 3D) multi-echo gradient echo acquisition with a water–fat separation reconstruction to extract spatially resolved fat fraction maps. The water and fat contribution to the signal within one voxel is determined after accounting for known confounding factors, including the presence of multiple fat peaks, T_2_^*^ decay, T_1_ bias, and phase error effects, as already shown in the liver (54) and bone marrow (60). However, there is no systematic analysis on the effect of confounding factors for BAT PDFF estimations using chemical shift encoding-based water–fat imaging. Thus, the methodologies already available for PDFF estimation in other tissues should be adopted to obtain confounder-free BAT PDFF estimation, such as using an average adipose tissue fat spectrum (61, 62), using small flip angles for reducing T_1_ bias effects (63, 64), and addressing phase errors depending on the employed imaging methodology (65, 66). If phase errors can be corrected, complex-based methods should be preferred over magnitude-based methods, thanks to the reduced sensitivity of complex-based methods to signal model mismatches (67, 68). If magnitude-based methods are employed, magnetic susceptibility-induced fat resonance shifts can affect PDFF quantification especially in the borders of the supraclavicular fossa (69). Finally, T_2_^*^ decay effects should be considered since BAT has shorter T_2_^*^ than WAT.

Just like MRS, chemical shift encoding-based water–fat imaging of the human supraclavicular fossa is challenged by strong macroscopic magnetic field inhomogeneities, which are particularly strong in the supraclavicular region, located right above the lungs, by variations in macroscopic field inhomogeneities due to respiratory motion, which make the magnetic field gradient time-dependent, as well as by microscopic magnetic susceptibility gradients. The presence of time-dependent magnetic field gradients can result in an incorrect field map estimation and produce the infamous water–fat swaps in the water–fat separation reconstruction. There is a plethora of field mapping techniques addressing such field map errors by imposing a smoothness constraint on the field map estimation (70) or by demodulating an a priori known magnetic field inhomogeneity contributions (71, 72).

### Z-Spectrum Imaging [Recently Proposed Technique]

An alternative method to chemical shift encoding-based water–fat imaging, named Z-spectrum imaging (ZSI), was recently proposed to measure tissue fat content. The method consists in sweeping a saturation pulse across a spectral region that includes water and fat frequencies. Signal intensity as a function of saturation frequency is then used to obtain a Z-spectrum from which water and fat signal components can be extracted, on a voxel-by-voxel basis. This method was initially tested in excised BAT samples and *in vivo* in humans (73), and later implemented to measure temperature in a water–fat emulsion phantom (74). With this method, the phase of the MR signal is completely ignored, so there are no phase distortions, and no assumption is made on the relative water–fat frequency, which is known to be greatly affected by microscopic field inhomogeneities in supraclavicular fat (69). Compared to PDFF, this approach is time consuming, as reconstruction of a Z-spectrum requires the acquisition of multiple images (one for each saturation frequency). Also, subject motion during the acquisition of multiple images, required for the Z-spectrum, may result in voxel misregistration and thus hamper the water–fat quantification.

### BAT Water–Fat Composition in Rodents

In chow-fed mice and rats living below thermoneutrality (i.e., standard laboratory living conditions), BAT is composed of multilocular adipocytes that are much smaller in size than white adipocytes. Brown adipocytes appear much more hydrated than classical WAT, with a water content typically ranging between 50 and 70%. In obese mice or humanized mice (mice living at thermoneutral conditions and fed a Western diet), the structure of BAT resembles much more that of adult humans, with UCP1-rich adipocytes surrounded by lipid-filled unilocular adipocytes, with much bigger cell sizes and with enlarged lipid droplets that often merge together into a single large lipid droplet (Figure 3) (75).

Detection of BAT water–fat composition in rodents by MRI dates back to 1989 (76), when chemical shift imaging was used in rats to identify not only the large iBAT depot, clearly visible in T_2_-weighted and T_2_^*^-weighted images, but also the much smaller BAT depots found in the supraclavicular, cervical, and axillary regions. In rodents housed under standard laboratory living conditions, which lead to chronic thermal stress in mice, these depots maintain the unique ~50/50 −30/70 water–fat composition characteristic of BAT (77), and therefore are easily identifiable on fat fraction maps. More interestingly, selective detection of water and fat signals enables not just a more accurate identification of these smaller BAT depots than T_2_-weighted and T_2_^*^-weighted images, but also the detection of changes in tissue fat content produced by cold acclimation or drug treatment. In this case, both MRS and chemical shift encoding-based water–fat imaging can be used as a valuable tool for monitoring lipid loss in BAT (due to both lipid oxidation and lipid export) during stimulation of non-shivering thermogenesis (78).

While selective detection and mapping of water and fat proton density can be time-consuming, chemical shift encoding techniques have the major advantage of being much faster (79), enabling mapping of water–fat tissue content in the entire body of small rodents in just a few minutes and with very high resolution, which is key for the reduction of partial volume effects and for the detection of other small BAT depots, whose size is often smaller than a few mm^3^ (57).

### BAT Water–Fat Composition in Humans: Studies at Thermoneutrality

Histological analyses performed on both human cadavers (1, 7) and biopsies (4, 6, 46) have shown that the amount and microscopic appearance of BAT in humans vary widely. In newborn humans, who rely on BAT thermogenesis to maintain their core body temperature, BAT is much more hydrated than in adult humans, with BAT areas made predominantly of typical multilocular BAT cells (80). In adult humans, BAT is more heterogeneous: brown adipocytes comprise both unilocular and multilocular fat cells, mostly found dispersed among white adipocytes. This heterogeneity found in adult humans, which is not recapitulated in infants or in rodents, leads to a wide variation in tissue water–fat composition.

In humans, BAT water–fat composition is predominantly measured by using chemical shift encoding-based water–fat imaging techniques. These techniques are widely available in clinical MR scanners and have shown great reproducibility and repeatability, independently of BAT metabolic activity (81–83). To detect and characterize BAT, chemical-shift encoding-based water–fat imaging techniques have been used on the supraclavicular adipose tissue in humans across all ages. Studies in infants (48, 84, 85), children (79), adolescents (86), and adults (81, 87–94) have demonstrated the feasibility of this technique in humans. A favored method to measure BAT fat fraction is the measurement of the PDFF, which has been used in several studies for human BAT on grounds of its robustness and standardization (81, 88, 92, 94). Histological verification of MRI results was only performed in small post-mortem studies (48, 84) and in a single case of a living human adult (95).

### BAT Water–Fat Composition in Humans: Studies During Cold Exposure

Cold activation induces lipid consumption in BAT. Several recent studies have tracked water–fat composition of supraclavicular fat in adult humans during cold activation. While some studies reported a decrease in supraclavicular fat fraction after cold-induced non-shivering thermogenesis, thus pointing to evidence of BAT lipid consumption in the region (90, 96–101), other studies did not find a consistent or significant change in fat content (34, 101).

The presence of two BAT cell populations, one with a higher lipid content that is predominantly consuming fat for thermogenesis, and one with a lower lipid content that is refueling its lipid stores from glucose and fatty acid uptake, could in part explain the contradictory results found in the literature (102). In support of this hypothesis, a recent study, which grouped the pixels into ranges of percentage decades and followed their changes over time (99), found a significant cold-induced decrease in lipid-rich voxels of the supraclavicular depot and a concomitant significant increase of PDFF in voxels with lower PDFF. Similarly, another study, which analyzed fat fraction on a voxel-by-voxel basis (102), found the largest cold-induced changes occurring in the fat fraction range of 70–100% at thermoneutrality (102). As the presence of two cell populations has never been reported in rodents, additional studies are needed to support this hypothesis.

Furthermore, while some studies have reported a correlation between glucose uptake in PET examinations and MRI fat fraction found after cold activation of BAT (98, 103, 104), other studies have found no correlation (34, 92, 101). In particular, by using combined PET/MR scans, McCallister et al. tried to find a difference in fat fraction between glucose avid and non-glucose avid regions within the supraclavicular fat depot. Despite some areas of very high glucose uptake appeared to display lower fat fractions in a couple of their study subjects (92), this was not the case in all of the subjects examined. Also, PET-based glucose uptake was shown to correlate with changes in MRI fat fraction in some, but not in all subjects (92). We believe that the greater BAT heterogeneity found in adult humans, which is not recapitulated in rodents that live under constant thermal stress, thus have well-defined and confined BAT depots, is the main reason for the conflicting results found in adult humans.

### MRI of BAT Water–Fat Composition in Metabolic Dysfunction

BAT has been shown to be involved in body weight regulation, glucose, and lipid homeostasis in mice (105–107). In humans, an increase in BAT activity, measured by PET/CT, has shown to be correlated with increased energy expenditure (104, 108, 109). Furthermore, an inverse correlation was found between BAT volume of activity and body mass index (BMI), as well as body adiposity (110, 111). These correlations are of interest regarding potential therapeutic targets for obesity. More interestingly, it has been shown that activation of BAT increases glucose and triglyceride clearance in humans, potentially decreasing the risk of developing diabetes and other obesity comorbidities (4, 86, 108, 112, 113). In human adults with obesity, effects of cold and insulin on BAT activity were reported to be restricted (114–116). MRI studies of BAT influencing metabolic dysfunction and secondary diseases have been performed more recently. A correlation between the supraclavicular PDFF, as a surrogate marker for BAT presence, and anthropometric as well as MRI-based obesity markers was shown (88). In adult patients with clinically manifest cardiovascular disease, an assumed presence of BAT, determined by MRI fat fraction in supraclavicular adipose tissue, correlated with a more favorable metabolic profile and less obesity (81). Male human subjects with obesity showed higher MRI-based fat fraction and fat fraction changes under thermal challenges correlated with hypermetabolic BAT volume and with BAT activity, as measured by PET/CT in this study (98).

### Limitations of Water–Fat MR Based Techniques

A clear identification of BAT only based on MRI fat fraction measurements remains challenging for several reasons. First, in rodents and humans, BAT lipid content is modulated by acute and chronic exposure to cold or adrenergic stimulation; thus, it varies widely with age, BMI, thermal living conditions, and diet (1, 7, 46, 75, 107, 117). Consequently, the water–fat composition of BAT can be a moving target, which alone can be limited in indicating the presence/absence of BAT.

Second, adult humans lack relatively large and homogeneous BAT depots such as the iBAT depot present in rodents. In humans, most BAT is localized in the supraclavicular fossa, which histologically is known to be a heterogeneous mixture of BAT and WAT. This heterogeneity is reflected in a heterogeneity in fat–water content in the region (6). In principle, one could think of increasing spatial resolution to reduce partial volume effect and become more “sensitive” to the cellular structure. In practice, however, a twofold reduction in the linear voxel dimension will require an increase in scan time of 64-fold, to maintain the same signal-to-noise ratio (SNR) (118). More importantly, fat fraction mapping techniques assume that each voxel encompasses the exact same anatomical region during spatial encoding, an assumption that can be easily violated when small voxels are employed in a region like the supraclavicular region, which is highly susceptible to motion.

Third, as adult humans are rarely under constant thermal stress, human BAT hydration is considerably reduced with respect to rodent BAT and presents a much higher intra- and inter-subject heterogeneity that can explain the different contrasting results reported in the literature. Specifically, while some measurements of PDFF in supraclavicular fat have shown a strong correlation between BMI and tissue fat content in the region (88), other studies trying to determine a fat fraction threshold to differentiate BAT from WAT within the supraclavicular fat region have reported that no universal fat fraction cutoff could be identified to reliably differentiate BAT from WAT (119). Another study using combined PET/MRI showed that, within the supraclavicular fat depot, glucose avid BAT regions had similar water–fat composition of glucose negative region, indicating that a subject-dependent fat fraction cutoff does not exist either (92).

Adipose tissue hydration has been reported to vary significantly, not only in regions containing BAT, but also in WAT (88, 120). Specifically, human subcutaneous adipose tissue PDFF has been shown to be positively correlated with anthropometric obesity markers (88). Although the exact explanation for the above association remains unclear, the variability of WAT hydration further complicates the use of PDFF for studying BAT in humans.

Even though many recent studies have been employing PDFF measurements addressing known confounding factors, there is a significant amount of literature reporting signal-weighted fat fraction values, which challenges the direct comparison of human supraclavicular fat fraction results across imaging studies. Therefore, standardization of fat fraction measurements in the human supraclavicular fossa would be beneficial.

Within the last 7 years of MRI studies using fat fraction techniques, it seems that fat fraction within the human supraclavicular fossa is not specific enough to identify pockets of BAT mixed with WAT within the supraclavicular fat in adult humans. While PDFF is a specific quantitative biomarker of hepatic steatosis, PDFF is not a specific biomarker of BAT. Nonetheless, measurements of changes in tissue fat fraction upon activation could increase PDFF specificity to BAT (38–40). In this context, it is important to point out that functional measurements including changes in tissue fat fraction or glucose uptake using PET may increase PDFF specificity. However, such functional measurements may not add any additional information in subjects in which BAT has a reduced fat-burning capacity or glucose uptake capacity at the time of measurement.

### MR Image Analysis Aspects

The main interscapular BAT depot is most commonly used for BAT characterization in rodents. Because of its homogenous and unique microstructure, this region can be easily segmented from muscle and subcutaneous fat. Previous efforts to automatize the segmentation procedure of interscapular BAT by MRI have been using neural network techniques applied on fat fraction and T_2_ measurements, as well as on fat fraction and T_2_ and T_2_^*^ measurements (121, 122).

Image analysis and segmentation of the human BAT are more challenging. As in rodents, the analysis is often restricted to areas where BAT is most commonly found, and which comprises the cervical, supraclavicular, and axillar region, where this tissue is found between muscles and surrounding major blood vessels. Segmentation of human BAT is challenging because:

1. There is no clear or consistent tissue boundary toward the cranial, caudal, or lateral direction.
2. Partial volume effects occur, even with an image resolution of 1 mm^3^:
  - Histological analysis of human supraclavicular fat has shown that the area contains only small clusters of multilocular brown adipocytes, mixed with WAT (1, 4, 75). At the typical resolution used in MRI, one should expect partial volume effects.
  - The pocket is rich in larger and smaller blood vessels containing blood that contribute to partial volume effects.
3. There is no consensus on the lower and upper fat fraction threshold needed to segment this tissue. In many human fat fraction studies, the entire supraclavicular fat, with a fat fraction ranging from 30 to 80%, is often labeled as BAT, with the lower boundary only needed to exclude muscle and blood vessels. Others have included all voxels with a PDFF greater than a chosen lower threshold.

The most commonly seen approach for BAT segmentation involves a crude manual segmentation in the supraclavicular fossa, followed by applying a threshold and an erosion step to prevent partial volume effects from neighboring tissues such as muscle.

Nonetheless, a semi-automated segmentation for processing BAT in the cervical–supraclavicular depot was suggested using an atlas-based approach (123). The atlas was generated based on previously manually segmented data and aimed at including adipose tissue located between the clavicle and the scapula. A manually pre-segmented data set was then registered onto the atlas to generate the final mask. However, not all of the adipose tissue between the bones was included and the lateral borders of the mask did not follow a clear anatomical structure. A different study, that had both cold-activated PET/CT data and MRI data available in over 20 subjects used information from all three image modalities to generate a common mask. A rigid image co-registration was done to overlay the MR data with the PET/CT data scans (89). In any case, special care is needed not to include neighboring bone tissue signal that also has fat fraction values similar to adipose tissue.

When PDFF techniques are used to characterize differences in fat fraction in supraclavicular fat over time, or after acute stimulation of BAT thermogenesis, co-registration of pre- and post-PDFF maps also needs to be addressed (102).

## Characterizing Bat Microstructure

### T_2_^*^ and R_2_^*^ Mapping at Rest [Widely Used Technique]

T_2_^*^ mapping quantifies the presence of microscopic field inhomogeneities and can be sensitive to microstructural components with different magnetic susceptibility. T_2_^*^ mapping is a popular imaging technique for measuring endogenous iron concentration in different organs (i.e., brain, liver) or the concentration of exogenous paramagnetic contrast agents. T_2_^*^ mapping is typically performed using a multi-echo gradient echo acquisition and it can be combined with a single T_2_^*^ decay signal model in tissues containing just water, and with the water–fat signal model in tissues containing both water and fat. BAT T_2_^*^ mapping has been therefore previously performed in combination with fat fraction mapping using a chemical shift encoding-based water–fat imaging method. Multiple previous works have shown that BAT has shorter T_2_^*^ and thus greater R_2_^*^ (defined as the inverse of T2^*^) than WAT, both in mice (124) and in humans (79, 90, 96, 98, 125–127). The shorter T_2_^*^ of BAT is attributed to the abundance of iron-rich mitochondria, which confer to this tissue its characteristic brown color.

**Figure 5:**
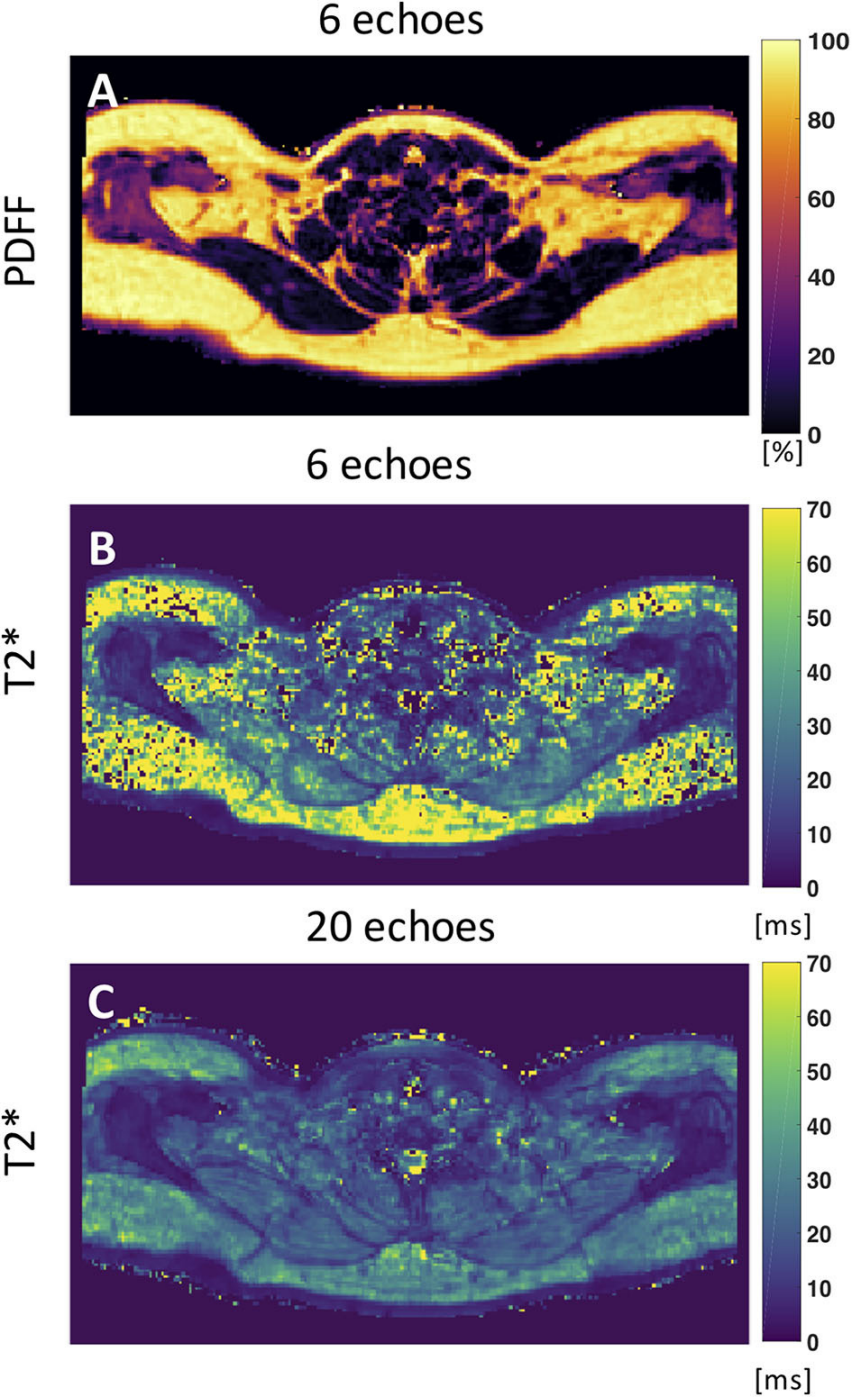
Axial PDFF and T_2_^*^ maps of human supraclavicular fat. **(A)** PDFF map calculated using 6 echoes. **(B)** The T_2_^*^ map estimated from the 6-echo data set is noisy and includes severely overestimated values in both the subcutaneous region and the supraclavicular fossa. **(C)** The T2* map estimated by using 20 echoes yields a more robust and smooth fitting result. This figure is original and based on data from (125).

Despite numerous publications on the accuracy and precision of T_2_^*^ mapping in water-dominant tissues, little is known about the accuracy and precision of T_2_^*^ mapping in fat-dominant tissues. A recent study showed a markedly decreased range of T_2_^*^ value and standard deviation of supraclavicular and gluteal adipose tissue when using a 20-multiecho gradient echo acquisition vs. a 6-multiecho gradient echo acquisition (125). One factor that contributes to the overestimation of T2^*^ in case of considering fewer echoes is that the exponential decay, caused by T2^*^, is not well-depicted when only considering the shorter echo times in the signal fitting routine. A higher number of sampled echoes at a constant echo time step (optimal for water–fat separation) has been therefore recommended in order to improve adipose tissue T_2_^*^ precision and to decrease the sensitivity of adipose tissue T_2_^*^ results on the underlying fatty acid composition (125) (Figure 5).

### Probing Microstructure With Intermolecular Multiple Quantum Coherences [Recently Proposed Technique]

Non-linear MR signals, such as those originating from intermolecular multiple quantum coherences (iMQCs) between water and fat spins, have been used to detect BAT. iMQCs between ^1^H spins residing in water and fat molecules can be made observable by using a clever combination of RF pulses and magnetic field gradients that modulate the longitudinal magnetization, break its spatial isotropy, and reintroduce the effect of long-range dipolar field interaction between the correlated spins (128, 129). Under the effect of the long-range dipolar field, antiphase magnetization originating from iMQCs can evolve in an observable signal. Modulation of the longitudinal magnetization on a cellular size scale can be used for amplifying the iMQC signal between water and fat spins that are only a few micrometers apart, while suppressing the iMQC signal between water and fat spins that are far from each and that possibly reside in different tissues. Therefore, these signals can provide a better way to probe water–fat composition at the cellular level, a scale that is currently inaccessible by clinical MR techniques.

The ability to select water–fat iMQC signal from BAT, and suppress water–fat iMQC signal from mixtures of fat and muscle, was first demonstrated in 2011 (130). Later, the same technique was applied *in vivo* to map BAT distribution in mice (118) and rats (131).

Applications in humans, however, have been limited by the poor SNR achievable with clinical scanners. The iMQC signal intensity scales as the square of magnetization density and thus as the square of the magnetic field strength. This means that at 3 T, the signal is only 20% of the signal obtainable at 7 T, at which mouse studies have been performed (118), and <4% of the signal obtainable at 9.4 T, at which rat studies have been performed (131). Also at 3 T, the dipolar demagnetization time (i.e., the time needed to the dipolar field to refocus the signal originating from iMQCs) is several hundreds of milliseconds—much longer than the characteristic T_2_ and T_2_^*^ of BAT, thus further limiting the detectable signal. Finally, the water–fat iMQC signal intensity is maximized for homogeneous equal emulsions of water and fat spins, on which these techniques are typically tested (132, 133). Thus, while at 3 T the low signal intensity may still enable low-resolution background free maps of BAT in humans, the relative scarcity and heterogeneity of BAT depots in humans, coupled with its less favorable water–fat composition, may preclude collection of high-resolution 3D maps needed for accurate quantification of BAT.

### Probing Microstructure via Diffusion Contrast

#### Diffusion-Weighted MR Measurements

Diffusion-weighted imaging (DWI) can probe water diffusion at the microscopic level. In DWI, magnetic field gradients are applied to encode and decode nuclear spin positions at the microscopic level, along a specific direction. In the presence of molecular diffusion between the encoding and decoding gradients, the spins acquire an additional phase that prevents their complete refocusing during acquisition, leading to a signal reduction. As the signal reduction is directly proportional to spin diffusion, the images will acquire a contrast that is diffusion-weighted.

In addition to a qualitative assessment of water diffusion, that is for instance sufficient to detect an acute ischemic stroke, quantitative mapping of diffusion properties can be done to quantify water diffusion that directly reflects the microstructural properties of tissue. For this task, an ADC map is generated by acquiring images at different diffusion gradients strengths/timing (*b*-values) before fitting a mono-exponential signal decay on a voxel-by-voxel basis. By probing the diffusion behavior of both water and fat components, DWI and DW-MRS techniques can leverage the difference in cellular structure between BAT and WAT to differentiate these two tissues.

#### DWI of Water [Recently Proposed Technique]

A study on 28 subjects comprising normal-weight and obese children compared the average ADC value of the water signal between the two cohorts using a multi-shot turbo-spin-echo-based sequence (134). In this study, no fat suppression was used and only two *b*-values were used for ADC calculation. No significant difference was found between the two groups before adjusting for pubertal status and gender. The slight tendency of a more restricted water diffusion behavior in the BAT of the obese cohort was explained with a reduced extracellular space due to the presence of large adipocytes. However, it is important to note that to selectively probe diffusion properties of water in BAT, suppression of all lipid signals is required, as previously done in bone marrow (135).

#### DW-MRS of Fat and Water [Recently Proposed Technique]

DW-MRS enables one to assess the diffusion properties of both water and lipid signals, without the need for signal suppression. The measured diffusion-weighted signal depends on the lipid diffusion properties, and on the shape, dimensions, and orientation of the restricted geometry in which fat molecules diffuse. These are known to be very different in BAT compared to WAT. In BAT, triglycerides form multiple small fat droplets, whereas in WAT, they form a single, large, unilocular fat droplet. Because fat diffuses 100 times slower than water, to probe lipid diffusion properties, strong diffusion encoding gradients are required, which may not be available in a clinical scanner. By applying high *b*-value gradients at variable diffusion times, one can detect differences in cell size using DW-MRS (136, 137). This idea has been applied *in vitro* on *ex vivo* BAT samples excited from the interscapular region using a preclinical MR scanner: a lower ADC was found in the WAT samples of rats on a high-fat diet compared to those on a normal chow diet. The lower ADC in WAT of mice with a high fat diet has been linked to lower diffusion coefficients in the presence of longer fatty acid chain lengths and more saturated fatty acids in the triglycerides. Assuming fat droplets of a spherical shape, fitting the MR signal for different *b*-values to analytical signal expressions can provide an estimation of the droplet sizes in BAT (138). In rodents, smaller radii were found for BAT of chow diet rats, which agreed with histological findings.

*In vivo*, lipid droplet size measurements in non-moving organs (e.g., leg bone marrow) have been demonstrated (137). However, motion-induced intra-voxel spin dephasing will yield additional signal attenuation, which in turn leads to an overestimation of fat diffusion properties. This includes scanner table vibrations (139) but also physiological motion, including respiratory motion and tissue deformation due to vessel pulsation. A flow-compensated, cardiac-triggered and respiratory navigator-gated DW-MRS sequence has been proposed to compensate for motion other than diffusion (140) when scanning the supraclavicular fossa in humans. However, the fact that a signal decay of the fat peaks already at low *b*-values was detected, where we would not expect diffusion effects of fat, indicates residual motion effects on the measured DW fat signals—even when using flow-compensated diffusion waveforms.

**Figure 6:**
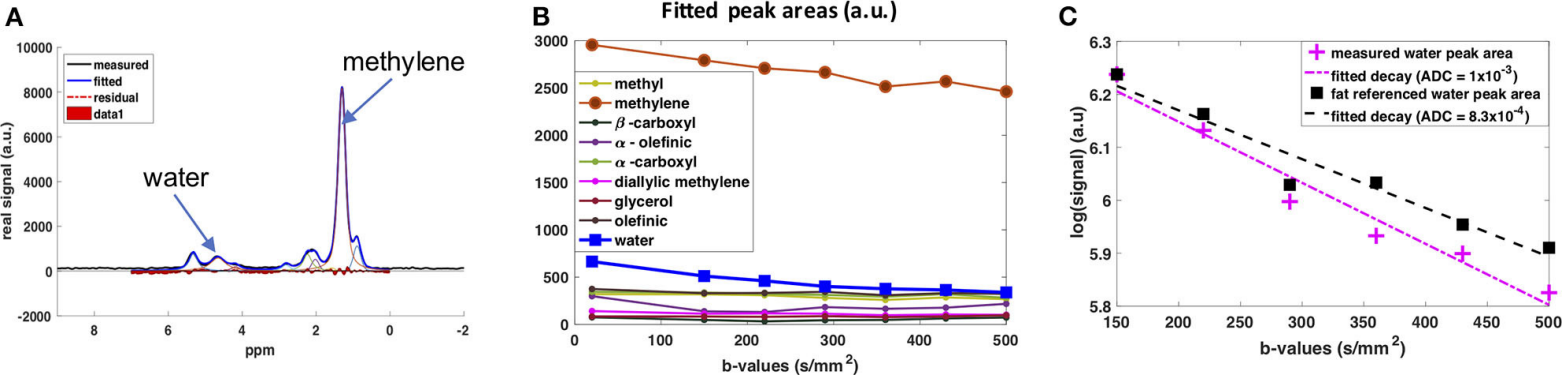
DW-MRS of water component in human supraclavicular fat using a both respiratory and cardiac triggered acquisition scheme. **(A)** Example of spectral fitting results for a voxel within the supraclavicular fossa. **(B)** Fitted spectral peak areas for eight fat peaks and water peak for a range of different *b*-values. **(C)** ADC fitting results for the water peak. In pink: Data points and fitted mono-exponential decay without fat referencing. In blue: Data points and fitted mono-exponential decay after referencing to the fat peaks, under the assumption that the decay in fat is due only to motion effects at these low *b*-values. This figure is original and based on data from (141).

The diffusion behavior of the water peak in the human supraclavicular fossa has been investigated as well (141). The acquisition was respiratory- and cardiac-triggered. The water signal was referenced to the fat peaks before fitting the ADC value to mitigate any residual impact of motion on the fitted ADC (Figure 6). This prevented an overestimation of ADC due to motion-induced intra-voxel dephasing. In this preliminary study, a more restricted diffusion behavior of the water signal in people with known brown fat was found (142), which stands in contrast to the findings of the study using DWI (134). A possible explanation for the more restricted water diffusion would be that a part of the measured water spins originates from inside the mitochondria of BAT, thus experiencing a more restricted environment compared to the cytoplasm or extracellular space in WAT. However, how cytoplasmic and extracellular water of BAT contribute to the DW-MRS signal is not clear. A complex mixture of hindered (by fat droplets and mitochondria) diffusion and restricted diffusion (by the cell membrane and the inner mitochondrial membrane) could be thought of for cytoplasmic water. Further measurements coupled with histological assessment of the tissue are required for a better modeling of the DW-MRS signal in BAT.

## Characterizing Bat Function

Beyond the commonly used ^18^F-FDG PET for measuring BAT function, MRI offers versatile contrast mechanisms to assess BAT function. The MR contrast mechanisms proposed to characterize BAT function and their application in differentiating BAT from WAT are reviewed in the next sections.

### Multi-Modal Imaging (PET-MRI) [Widely Used Technique]

**Figure 7:**
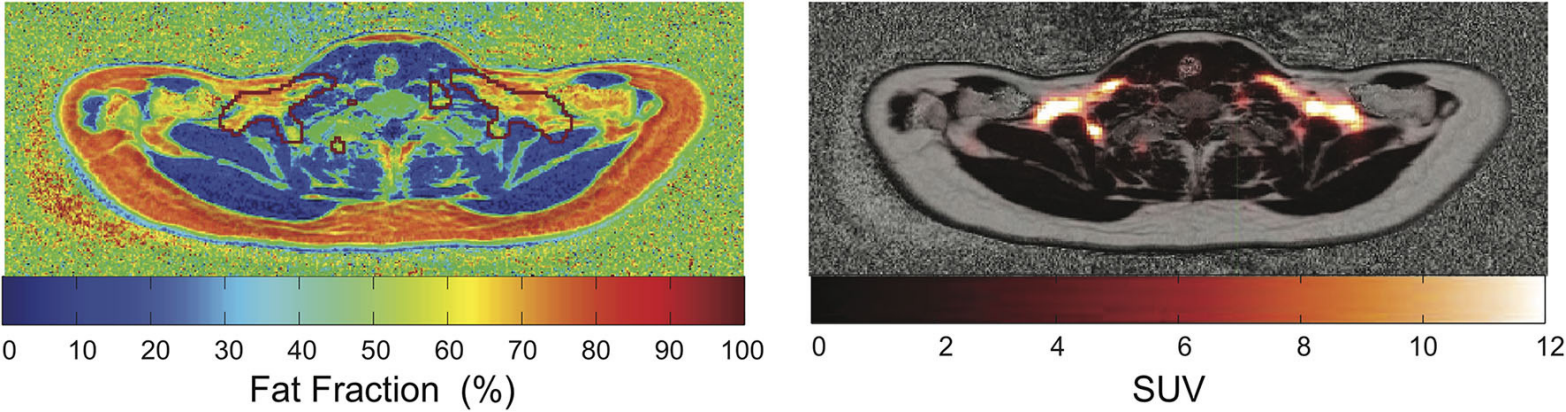
Overlay of PET images and MR-based PDFF maps. The red mark indicates regions with SUV > 2.5. This figure is original and based on data from (92).

While most of the human BAT studies are performed on PET/CT platforms, more and more studies are performed using PET/MRI. Major advantages of PET/MRI over PET/CT include a lower radiation burden (as MRI replaces CT for attenuation correction and anatomical information), and the possibility to better characterize glucose avid BAT regions in terms of iron content and tissue perfusion by MRI. When compared to MRI alone, the combined use of PET/MRI increases the accuracy of MRI BAT detection, at least in those subjects that exhibit metabolically active BAT in ^18^F-FDG-PET scans (Figure 7).

While several studies have shown that fat fraction in supraclavicular fat is significantly different than fat fraction of subcutaneous white fat (82, 119, 143), PET/CT and PET/MRI images of human BAT, as well as histology examinations of human BAT, have clearly shown that not the entire supraclavicular/cervical fat depot, but only a fraction of it contains BAT. Therefore, one logical question is whether fat fraction mapping techniques can help identify BAT within the much larger supraclavicular fat depot. To answer this question, PET/CT studies in combination with MRI studies (119, 126, 143) or combined PET/MRI studies (92) have been performed in the last 10 years. In these studies, it was clearly shown that significant differences exist in T_2_ and T_2_^*^ relaxation times, as well as in fat fraction values between subcutaneous and supraclavicular fat, but that the fat distribution of the supraclavicular/cervical fat compartments does not correlate with the FDG uptake. In other words, a specific fat fraction threshold could not be found *in vivo* in adult humans to differentiate areas with or without BAT within the supraclavicular fat depot. This finding should not be surprising given the large difference in BAT morphology and hydration between humans and rodents (75), in which most of the fat fraction methods have been validated.

Indirect evidence of the improved accuracy provided by combined PET/MRI technique is the ability to detect small changes in BAT fat fraction during prolonged cold exposure, at least in some subjects. MRI studies assessing the change in fat fraction and T2^*^ values of the supraclavicular fat depot upon activation have shown contrasting results on the correlation of these values with glucose uptake (126). However, a much higher correlation was found when the analysis was focused on selected regions within the supraclavicular fat. Although the degree of glucose uptake in each subject cannot be taken as a surrogate measure of the degree of BAT thermogenic activity, an increased glucose uptake is still expected to be present in functional BAT. Therefore, hybrid PET/MRI might represent the ideal multi-modal imaging tool for BAT evaluation.

### Metabolic Imaging With X-Nuclei

#### ^31^P, ^13^C, and ^2^H MR Measurements [Recently Proposed Techniques]

With ^31^P NMR spectroscopy, one can typically probe phosphate metabolism, which permits the phosphorylation of ADP, converting it into ATP. In ^31^P spectra, resonance frequency lines from free phosphate, phosphocreatine, as well as the α-, β-, and γ-ATP (where the Greek letter corresponds to the position of the phosphate within the ATP molecule) can be easily identified, while the concentration of ADP, under physiological conditions, is too low to be detected, and is typically calculated indirectly using the other ^31^P peaks. When evaluating ^31^P NMR spectra, a reduction in the phosphocreatine peak or in the PCr/ATP ratio is typically associated with an increase in cell energy demand, while the rate of PCr recovery is considered an indicator of mitochondrial oxidative capacity, thus providing a way to evaluate mitochondrial function *in vivo*. Interestingly, in a study investigating brown fat tissue dynamics by ^31^P NMR, no changes were observed in the PCr/ATP ratio (calculated as PCr/γ-ATP ratio), or in the ATP/ADP ratio (calculated as β-ATP/α-ATP ratio) (78), neither in wild type nor in UCP1-KO mice, and independently of previous cold or 30°C acclimation. This finding was interpreted as ATP still being generated at a sufficient rate during BAT activation to cover the cellular needs for enzyme activation and biosynthesis. When data of wild type and UCP1-KO mice before NA injection were pooled, the authors found a significantly lower PCr/ATP ratio in cold-acclimated mice compared to warm-acclimated mice, indicating a higher ATP concentration in BAT under chronic stimulation.

Although ^31^P-MRS has been used to study tissue metabolism *in vivo*, this technique is challenged by the low MR sensitivity to the ^31^P nucleus and the low *in vivo* concentrations of phosphate metabolites. Taken together, these two effects greatly limit spatial resolution and introduce confounding factors in the interpretation of the spectra from partial volume effect.

**Figure 8:**
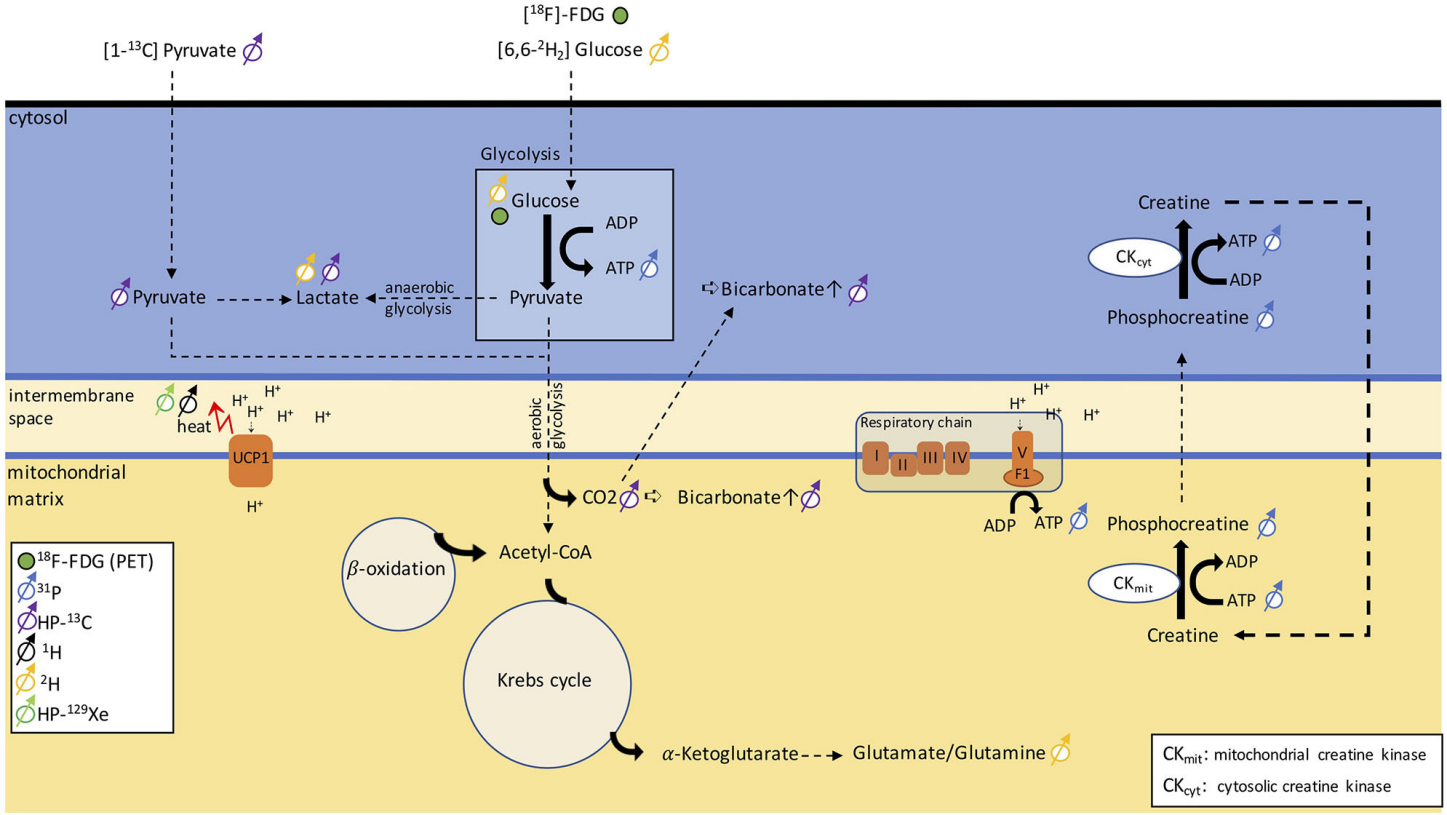
Schematic figure illustrating different metabolic pathways detectable with NMR. The uptake in glucose can be traced via PET using [^18^F]-FDG. ^31^P can be used to monitor ATP production and phosphocreatine metabolism. By injecting [1-^13^C]-pyruvate, we can follow anaerobic glycolysis via the conversion of pyruvate into lactate or aerobic glycolysis via the detection of a bicarbonate signal. Alternatively, [6,6-^2^H]-glucose can discern anaerobic from aerobic glycolysis by detecting lactate and the glutamate/glutamine peak. The heat generated in BAT can be detected as a shift in resonance frequency of both water ^1^H and lipid-dissolved HP-^129^Xe.

^13^C is a stable isotope of carbon. Unlike ^12^C, the most abundant stable isotope of carbon with a zero nuclear spin, ^13^C has a nuclear spin of ½ and is therefore MR-visible. Because of the low natural abundance of this isotope, *in vivo* ^13^C MRS is technically and experimentally challenging and typically requires infusion of ^13^C-labeled molecules. Hyperpolarization increases the MR sensitivity to the ^13^C-labeled molecules by four orders of magnitude, such that one can detect these molecules *in vivo* at micromolar concentrations and track their metabolic conversion into their downstream metabolites. Among the different hyperpolarized (HP) ^13^C tracers, [1-^13^C]-pyruvate is most widely used because it is easy to polarize and it has a relatively long longitudinal relaxation time (T_1_ ≈ 60 s), which enables the detection of this molecule and its downstream products before the nuclear spin polarization decays back to thermal equilibrium. In tissues, conversion of HP [1-^13^C]-pyruvate into [1-^13^C]-lactate, [1-^13^C]-alanine, and [^13^C]-bicarbonate can be observed by ^13^C NMR spectroscopy and imaging right after infusion of HP [1-^13^C]-pyruvate. The ratio between bicarbonate and lactate can be used as a marker of aerobic versus anaerobic metabolism (Figure 8), thus providing additional metabolic information on the fate of glucose that ^18^F-FDG-PET cannot provide.

While HP [1-^13^C]-pyruvate has been mainly used for preclinical and clinical cancer studies, two preclinical studies have shown applications of HP [1-^13^C]-pyruvate for BAT detection in mice and rats. In a study on cold-exposed mice (144), an increase in pyruvate tissue uptake and a non-statistically significant increase in the lactate/bicarbonate ratio was observed in cold-acclimatized mice compared to warm-acclimatized mice, suggesting a tendency toward increased anaerobic metabolism during cold exposure, contrary to what one would expect in BAT. Furthermore, it was found that the [1-^13^C]-bicarbonate/[1-^13^C]-pyruvate ratio increased by 13-fold after cold exposure, compared to the thermo-neutral condition. Yet, no differences in pyruvate metabolism were observed during acute noradrenergic stimulation of BAT, in marked contrast to an early study that had shown increased conversion of pre-polarized [1-^13^C] pyruvate into ^13^C-bicarbonate and [1-^13^C]-lactate in rats (145). This discrepancy between the two studies, however, should come as no surprise. While in the earlier study, rats were anesthetized with ketamine, in the second study, mice were anesthetized with isoflurane, an anesthetic known to suppress BAT thermogenesis and blunt BAT response to noradrenergic stimulation.

Also, one should keep in mind that quantification of anaerobic/aerobic metabolism with [1-^13^C] pyruvate in BAT is challenging, as this requires mapping of [1-^13^C] pyruvate and its downstream products at a relatively high spatial (to avoid partial volume effects) and temporal resolution, to enable kinetic analyses. Clearly, the reduced intensity of the bicarbonate peak (~2 orders of magnitude smaller than pyruvate), coupled with the limited spatial/temporal resolution with which dynamic metabolites data can be acquired, makes quantification of aerobic/anaerobic metabolism in BAT a challenge.

Deuterium, ^2^H, is a stable isotope of hydrogen with a very low natural abundance (<2%) and with a nuclear spin of 1. The short longitudinal relaxation time of this quadrupolar nucleus enables rapid signal averaging and the acquisition of a deuterium spectrum from natural abundant deuterium in a matter of several minutes, at high magnetic field strengths. The deuterium chemical shift range is comparable to that of ^1^H. Therefore, without injection of a ^2^H-labeled tracer, deuterium spectra of tissues closely resemble ^1^H spectra. Because of the low natural isotopic abundance of ^2^H, ^2^H-NMR spectroscopy is often performed in combination with oral intake or intravenous infusion of non-radioactive ^2^H-labeled glucose, often used to assess tumor metabolism. Injection of [6,6-^2^H_2_]-glucose at mM concentrations leads to the appearance in the ^2^H spectra of a glucose peak whose intensity is comparable to that of natural abundance ^2^H water. After several minutes, conversion of ^2^H-labeled glucose into downstream metabolites such as glutamine and glutamate, as well as lactate (Figure 8), has been observed *in vivo* in ^2^H spectra of the brain and liver. Thus, this technique, named deuterium metabolic imaging (DMI), as ^13^C, can reveal glucose metabolism beyond mere uptake.

Recently, a study demonstrated the application of DMI to study BAT in rats at 9.4 T. In this study, a rapid increase followed by a rapid decrease of the injected ^2^H-glucose signal was observed in cold-acclimated rats but not in warm-acclimated rats, whereas the lactate/glucose ratio was not significantly different between the two groups. When looking at the spectra, however, spectral resolution and SNR seem to be a major issue for quantification. While in the brain, good field homogeneity and the lack of a lipid signal are a prerequisite for robust quantification of glutamate/glutamine and lactate, in lipid-rich BAT, the presence of a relatively large lipid signal at about 1.3 ppm from natural abundant ^2^H lipids (which is typically much larger than the lactate signal originating from injected ^2^H-labeled glucose) is expected to make quantification of ^2^H-labeled lactate at 1.33 ppm a challenge. Surprisingly, no lipid peak contaminations were reported in the DMI BAT study by Riis-Vestergaard et al. (146), despite the high fat content typically present in the BAT of rats. Aside from the lipid contamination, which can be a major confounding factor in the interpretation of DMI spectra of fat tissues, it is important to note that ^2^H SNR scales as the square of the magnetic field strength; thus, high magnetic field strengths will be paramount for human translation of this technique.

### Measuring BAT Perfusion

While in general tissue perfusion does not always match tissue metabolism (147, 148), in both rodents and humans, sympathic nervous system stimulation of BAT typically results in an increase in tissue perfusion (17, 149–152).

During stimulation of thermogenesis in BAT, local vessels and capillary beds are dilated to increase BAT perfusion, while arterial–arterial shunts are used to divert blood from WAT to BAT (153). However, because the adrenergically stimulated increase in BAT blood flow is qualitatively and quantitatively independent of thermogenesis (148) [increase blood flow to BAT is observed in mice regardless of the degree of their thermogenic response (41, 154)], one should keep in mind that measurements of changes in BAT perfusion represent a promising way to probe BAT stimulation but not thermogenic response in BAT.

#### Contrast-Enhanced MRI [Recently Proposed Techniques]

The standard procedure in routine clinical MRI for assessing tissue perfusion relies on observing the local signal enhancement after intravenous injection of a contrast agent, such as a gadolinium compound, which shortens T_1_ relaxation. Therefore, T_1_-weighted sequences are employed for assessing perfusion using dynamic contrast-enhanced MRI (DCE-MRI). By tracking the MR signal dynamics during the time after contrast agent injection, the time-to-peak or the mean transit time can be calculated for a given anatomical region.

Sbarbati et al. (155) studied the interscapular BAT of rats after BAT activation with adrenaline and compared the spin echo-based DCE-MRI signal to the same signal from a control group. In contrast to the skeletal muscle of the anterior limb, the signal enhancement in the iBAT region was found to be significantly higher for the stimulated group compared to the non-stimulated group. Similarly, another study in rats used DCE-MRI in a cold-exposed and a thermoneutral control group (156). The uptake of contrast agent was three times higher in the iBAT of the activated group compared to the control group, while no significant difference was found in the interscapular WAT. More recently, a more quantitative study of the uptake kinetics using DCE-MRI, correlated with fat fraction maps, was performed in rats (157). The volume transfer constant from blood plasma into the extracellular-extravascular spaces and the concentration of the contrast agent were calculated. The iBAT depot was studied after cold activation and compared to a thermoneutral group. Activation of the iBAT depot was also induced by injection of a β_3_-agonist and compared to a control group injected with saline. Moreover, the effect of beiging of inguinal WAT after chronic injection of a β_3_-agonist was demonstrated. The study showed a decrease in fat fraction, and an increase in tissue perfusion in both iBAT and inguinal beige fat after activation.

Because of the risks associated with gadolinium injection (nephrogenic systemic fibrosis and the yet unknown consequences of long-term gadolinium depositions in tissues such as the brain), DCE-MRI has not yet been used in humans to study BAT. However, a rich blood perfusion of human BAT was reported as an incidental finding during MR angiography in newborn children and shown in previous review publications (39, 40).

An alternative to T_1_-shortening gadolinium-based contrast agents are superparamagnetic iron oxide nanoparticles (SPIONs), that shorten T_1_, T_2_, and T_2_^*^. Monocrystalline iron oxide nanoparticles (MIONs) belong to the group of SPIONs that have a long blood pool half-life and are already clinically used for liver imaging and angiography. MIONs have been used to study BAT perfusion in rats after injection of a β_3_-adrenergic receptor agonist (158). Blood perfusion was estimated based on the signal intensity change in T_2_-weighted fast spin-echo images. However, a low accuracy was reported for this method.

Radioactively labeled superparamagnetic iron oxide nanoparticles [Triglyceride-Rich Lipoprotein (TRL)-^59^FE-SPIOs] have been embedded into a lipoprotein layer to study the uptake of the nanoparticles by different organs, such as the liver, blood, muscle, and BAT after either intravenous or intraperitoneal injection (159). Cold-exposed mice showed a significant decrease of T_2_^*^ in BAT while this was not observed in the control group. Irrespective of the BAT activation, the TRL-^59^FE-SPIOs led to a decrease of T_2_^*^ in the liver, and no significant uptake was detected in the muscle. Radioactivity measurements revealed quantitative distributions of the lipoproteins in the respective organs.

#### Arterial Spin Labeling [Recently Proposed Technique]

Arterial spin labeling (ASL), also known as arterial spin tagging, has been extensively used to study brain perfusion without the need for contrast agent injection. In ASL, nuclear spins in an artery are first tagged by using either an inversion pulse or saturation pulse. When the tagged blood reaches the perfused capillaries further downstream, the contrast in the now acquired image changes depending on the rate of perfusion. A small human study conducted by Dai et al. found an increased perfusion of 86 ± 32% in BAT after cold exposure in a cohort of 10 young volunteers, but claim that this evaluation is challenging due to the proximity to large vessels, which leads to an overestimation of the perfusion (160).

#### Hyperpolarized Xenon MRI [Recently Proposed Technique]

Hyperpolarized xenon gas-enhanced MRI, a technique widely used to detect lung ventilation function in humans, has also been used to detect BAT, both in rodents (41, 154) and in humans (161). Xenon gas has a relatively high solubility in tissue (162, 163). This means that, once inhaled, the inert gas slowly diffuses into tissue and blood. Because of the high solubility of xenon in lipids (162), circulating xenon preferentially accumulates in fat-rich tissues, like BAT, at a rate that is directly proportional to tissue perfusion and blood flow. As a result, upon xenon inhalation, changes in BAT blood flow can be detected easily in xenon MR spectra and in images as an increase in the lipid peak originating from xenon dissolved in the lipid compartments of BAT (41). Because the degree of xenon uptake in BAT depends not only on tissue blood flow, but also on xenon solubility in the tissue (i.e., tissue fat content), similar signal enhancements are typically observed in BAT of obese mice, in which vascular rarefaction (BAT adipocytes are in general much larger than in lean mice) and the consequent reduction in tissue blood flow is overcompensated by the increase in xenon solubility in the tissue.

**Figure 9:**
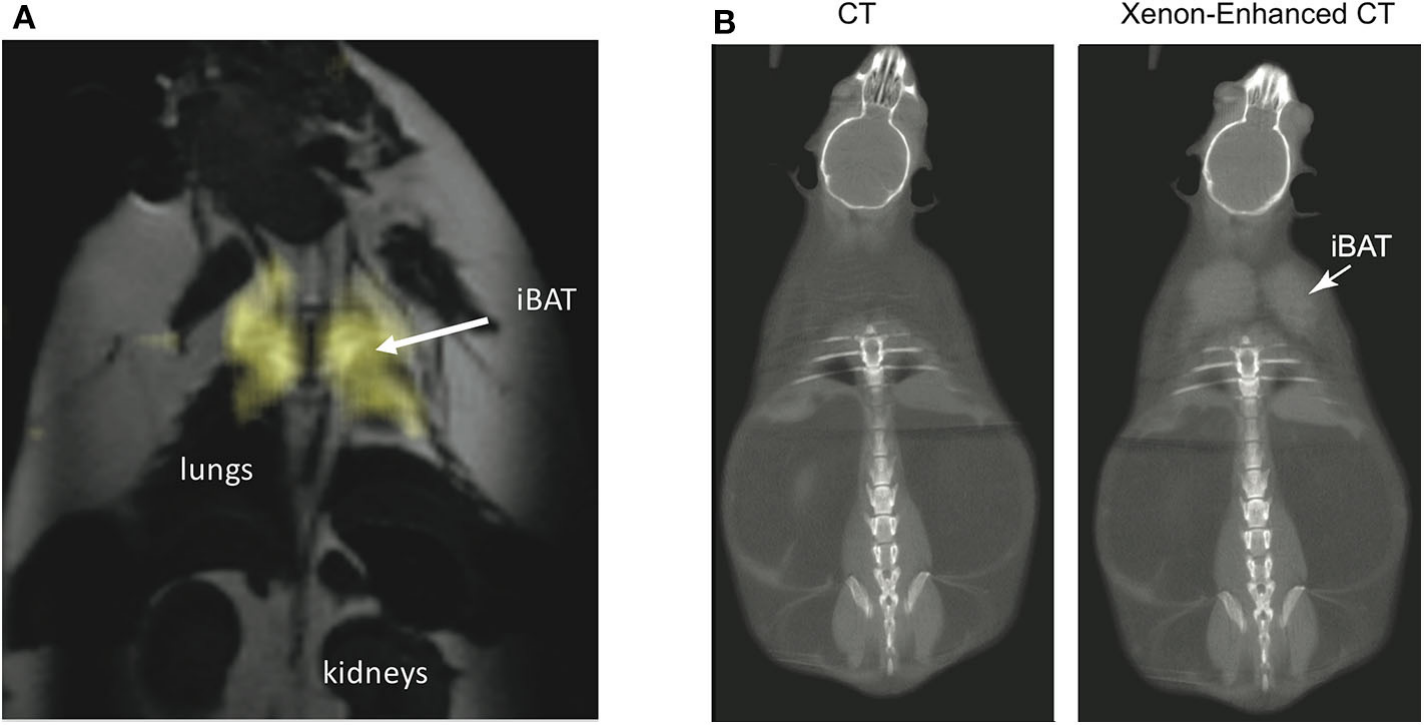
Xenon uptake in BAT of an ob/ob mouse detected by hyperpolarized ^129^Xe MRI and xenon-enhanced CT. **(A)** 2D HP ^129^Xe MRI (yellow) overlaid onto an anatomical ^1^H image. The HP ^129^Xe image shows background free maps of iBAT in an ob/ob mouse. This figure is original and based on data from (41). **(B)** (Non-enhanced) and (xenon-enhanced) coronal CT image of a different ob/ob mouse. Selective xenon uptake in iBAT leads to a remarkable change in tissue radiodensity. This figure is original and based on data from (153).

By having a radiodensity similar to that of iodine, the same selective uptake of xenon into BAT during stimulation of BAT thermogenesis can be detected in CT images (153) with a spatial resolution greater than what is currently achievable with hyperpolarized xenon gas MRI (Figure 9). This was recently demonstrated both in rodents and in non-human primates, in which BAT microstructure and distribution more closely resembles that in humans (153).

While the specific and strong increase in xenon uptake in BAT can lead to background-free detection of BAT in HP ^129^Xe MR images, spatial resolution is not enough for accurate quantification of its mass, which in turn could be quantified by high-resolution xenon-enhanced CT (153). However, the strong temperature sensitivity of the chemical shift of xenon dissolved in the lipids of BAT can be used to directly probe tissue thermogenic function.

### Measuring BAT Oxygenation

#### T_1_ Relaxation [Recently Proposed Technique]

One recent work has suggested a combined chemical shift T_1_ mapping sequence that simultaneously maps T_1_ of fat and water in voxels containing both species to directly measure BAT oxygenation *in vivo* (164). For this study, a saturation recovery sampling scheme was implemented in combination with a phase-modulated readout. By using this approach, T_1_ values of water/fat emulsions equilibrated with different gas mixtures were measured along with the T_1_ of lipids in BAT *in vivo*, in anesthetized rats, under a carbogen (95% O_2_-5% CO_2_) challenge. In both cases, a decrease in lipid T_1_ was found with increased tissue oxygenation. In the analysis, a signal model consisting of a single fat peak was used, while in a similar work, a peak-resolved T_1_ estimation using an inversion recovery-prepared MRS sequence was employed to estimate the effect of oxygenation on T_1_ of the methylene peak of WAT and lard (165). Unfortunately, these two studies do not allow us to draw any conclusion on the T_1_ behavior of lipids in BAT during normal BAT activation for two reasons: First, it is not clear whether, under normal breathing conditions and during BAT activation, lipid oxygenation in BAT will increase or decrease. This is because, during BAT activation, while blood flow increases, oxygen consumption increases even more. Indeed, at least in mice, activation leads to a complete deoxygenation of venous blood, and to an overall increase in tissue R_2_^*^ and magnetic susceptibility (166, 167). Second, as temperature is also known to have a strong effect on the T_1_ of lipids (lipid T_1_ increases with temperature) (168), application of this technique to BAT would require simultaneous and independent measurements of both temperature and tissue oxygenation.

### Measuring Hemoglobin Dynamics in BAT

In contrast to the diamagnetic properties of water and fat, oxyhemoglobin is only weakly diamagnetic, while deoxyhemoglobin is strongly paramagnetic. Activation of BAT is expected to lead to changes in tissue perfusion as well as to changes in oxy and deoxyhemoglobin concentration in blood that can be observed as changes in T_2_^*^ as well as changes in magnetic susceptibility.

#### T_2_^*^-Weighted Imaging [Widely Used Technique]

Functional magnetic resonance imaging (fMRI) is based on the blood oxygenation level-dependent (BOLD) contrast (169). In activated areas of the brain, the increase in oxygen consumption and thus the increase in oxygen demand are overcompensated by an increase in tissue perfusion, which locally leads to a surplus of oxyhemoglobin in comparison to the strongly paramagnetic deoxyhemoglobin (170, 171). This leads to a reduction of the local susceptibility gradients, to a longer T2^*^, and thus to an increase in the MR signal intensity (172).

Conversely in BAT, activation leads to a *decrease* in blood oxygenation level and thus to a drop in T_2_^*^, and to a consequent reduction in MR signal intensity that can be clearly observed in rodents near the Sulzer's vein during noradrenergic stimulation of the tissue (166, 167). In humans, whether the MR signal increases or decreases upon activation is still unclear. While some studies have found an increase in the MR signal intensity in glucose-avid regions of the supraclavicular fat depot during cold exposure (158), other studies have found glucose-avid regions in which the signal increases along with regions in which the signal decreases (143). This is not surprising, as even in the brain, variations in the subject's heart rate and breathing pattern are known to result in significant MR signal changes that can be easily misinterpreted as the result of hemodynamic changes due to a local increase in metabolic activity (173).

#### Quantitative Susceptibility Mapping (QSM) [Recently Proposed Technique]

QSM aims at finding the underlying magnetic volume susceptibility values for each pixel based on the measured field map, which represents the off-resonance frequency with regard to the excitation RF frequency. Due to the iron-rich mitochondria in BAT, QSM could be a promising method to discern BAT from WAT. However, so far, only studies measuring the increase in BAT perfusion, and the relative change in magnetic susceptibility, during BAT activation have been reported (167). Specifically, a strong shift toward higher magnetic susceptibility values was detected during BAT activation in a mouse model after injection of a β_3_-agonist (167), in agreement with previous BOLD studies showing an increase in tissue deoxy-hemoglobin content and a consequent decrease in the MR signal intensity during BAT activation in mice (166, 167).

### Measuring BAT Temperature

#### 1H-Based MR Thermometry [Recently Proposed Technique]

BAT temperature measurements represent the most direct and accurate way to detect BAT metabolic activity as, when active, this tissue oxidizes fatty acids to generate heat (11). Yet, up to recently, non-invasive monitoring of thermogenic activity in rodents has been assessed only indirectly, by either measurements of resting energy expenditure with indirect calorimetry, by rectal temperature measurements, or by estimating the ability of the animal to defend its core body temperature when exposed to cold. The interpretation of these results is not obvious, as these measurements cannot differentiate the contribution of cold-induced non-shivering thermogenesis generated from BAT from that of cold-induced shivering thermogenesis generated from skeletal muscle. In humans, surface skin temperature measurements and whole body core temperature measurements have been performed (97), which present the same limitations found in rodents. Skin temperature measurements by infrared thermography are often used outside the MR scanner to detect BAT activation (104, 174). However, these measurements are not very specific as they are strongly influenced by other physiological responses such as vasoconstriction and consequent reduction in local tissue blood flow, as well as by the subcutaneous fat layer thickness, which makes these measurements even less reliable in overweight and obese subjects (175). MRI could play a pivotal role in assessing BAT thermogenesis. MRI has long been used for non-invasive temperature measurements of tissues as almost all MR parameters [T_1_ (176), T_2_ (177), proton density (178), diffusion (179), and water resonance frequency (179)] are temperature sensitive and have been used as tissue temperature sensors. In practice, in most preclinical and clinical applications, the temperature-induced proton resonance frequency shift (PRFS) of water is the preferred temperature probe for three main reasons: First, the temperature dependence of the water chemical shift is tissue-independent (−0.01 ppm/°C), removing the need of a pre-calibration scan in the tissue of interest; Second, the temperature dependence of water chemical shift is linear, further simplifying the conversion of frequency shifts into temperature changes; Third, this method is very fast as the water resonance frequency shift can be directly deduced from a phase change of the MR signal.

Unfortunately, the need of two images to extract frequency changes and the relatively small effect of temperature on the water resonance frequency make these measurements highly susceptible to motion; this encompasses tissue displacement of the ROI as well as field changes due to motion outside the ROI.

On a standard 3-T MRI system, the temperature-induced shift of the water resonance frequency is only about 1 Hz/°C, compared to the much stronger shift of 10–50 Hz induced by magnetic susceptibility gradients, field drift, and respiratory motion. While in tissues containing fat, the almost temperature-insensitive resonance frequency of lipid protons could, in principle, be used to correct for macroscopic field inhomogeneity and motion (180), in practice, this is not possible without incurring in temperature errors of a few degrees Celsius (181, 182). At the macroscopic and microscopic level, water and fat spins reside in different tissue compartments with different magnetic susceptibilities. Therefore, the local magnetic field experienced by water protons is not the same as the one experienced by fat protons. The difference in the local field experienced by the two chemically different spins, which depends on the specific intra-voxel distribution of water and fat spins and on the orientation of the distribution with respect to the main magnetic field, can be on the order of a few tenths of ppm (181–183). These microscopic susceptibility gradients preclude the possibility of a pre-calibration of water-fat frequency shift as a function of temperature *in vitro*, in samples that do not necessarily reflect the specific intra-voxel water-fat distribution and orientation found *in vivo*. In addition, the magnetic susceptibility of fat increases with temperature by 0.008 ppm/°C, leading to a change in the local magnetic field distribution that is typically observed as a broadening of the water resonance frequency peak, and to a non-linear and nonlocal relation between temperature and water resonance frequency (183). As already stated, the relative water–fat frequency separation strongly depends on the specific intra-voxel water–fat distribution. Therefore, *in vitro* calibration on water–fat mixtures cannot be used to estimate absolute tissue temperature *in vivo*, as it has been done in some studies (91). Also *in vivo*, voxel misregistration due to breathing motion can undermine measurements of temperature change in the tissue (184). While these effects due to the presence of fat have not precluded the use of PRFS for temperature monitoring during thermal ablation treatments of tumors, where the temperature is raised by tens of degrees Celsius to cause tissue necrosis (185), or in rodents, where BAT temperature can increase by more than 5°C (186), it can be a limiting factor in the measurement of the small temperature increase (1–2°C) expected in human BAT during cold exposure.

In principle, one could think of using iMQC to remove the effect of magnetic field inhomogeneity and motion at the microscopic level. One particular type of iMQC signal, the intermolecular zero quantum coherence (iZQC), evolves at the difference in frequency between water and methylene protons, resulting in removal of some inhomogeneous broadening at the microscopic level, and possibly enabling absolute temperature measurements in the tissue (132). In practice, this is precluded by the much higher sensitivity of the water–fat iZQC frequency to the specific water–fat distribution at the microscopic level (187).

#### 129Xe-Based MR Thermometry [Recently Proposed Technique]

**Figure 10:**
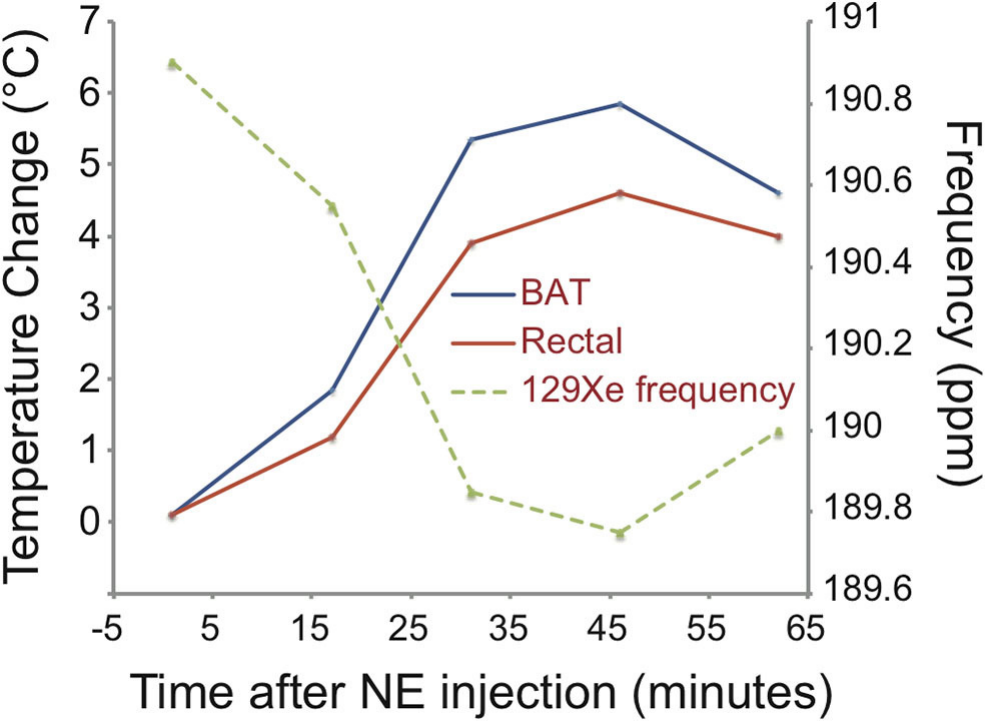
Temperature change in iBAT as detected by hyperpolarized ^129^Xe MR thermometry in a C57BL/6 mouse during norepinephrine stimulation of BAT thermogenesis. Right after the injection of norepinephrine, an up-field drift of the temperature-sensitive resonance frequency of xenon dissolved in the lipid droplets of BAT is observed. The frequency drift reflects a change in iBAT temperature of almost 6°C. The change in rectal temperature follows, with some delay, the change in BAT temperature, indicating that BAT is the main driver for the increase in body temperature. This figure is original and based on data from (41).

Recently, the use of hyperpolarized ^129^Xe gas for MR thermometry measurements in fat-containing tissues was demonstrated (188). This method relies on the much higher temperature sensitivity (−0.2 ppm/°C) of the chemical shift of xenon dissolved in fat, the major resonance frequency detected in ^129^Xe spectra acquired in BAT. This methodology was demonstrated in both rodents (41, 154, 183) and humans (189). More recently, the ability to directly measure absolute temperature in fat tissues was demonstrated (154). This is accomplished by using the lipid protons, in which xenon dissolves, as an internal resonance frequency reference to remove the effect of magnetic susceptibility gradients and to make the measurement insensitive to changes in the local magnetic susceptibility caused by possible changes in tissue oxygenation levels (Figure 10). Absolute temperature measurements would be particularly advantageous in humans, where a single measurement of absolute temperature could be used to tell apart active from inactive BAT.

## Discussion

MRI measurements of tissue fat fraction have gained much attention as a radiation-free alternative to ^18^F-FDG-PET/CT. Table 2 summarizes a critical assessment of existing MR methods in BAT research. These techniques have been proposed to differentiate the more hydrated BAT from WAT independently of BAT thermogenic activity and were the first to be used in humans. One reason is that MR fat fraction measurements are widely available on clinical scanners, where they are typically employed for estimating fat fraction in organs such as the liver, skeletal muscle, and bone marrow. However, as discussed in the 2019 ISMRM Workshop on MRI of Obesity and Metabolic Disorders (191), there is no consensus on the specificity of these techniques for BAT detection, or on the specific protocol needed for quantitative measurement of fat fraction and fat fraction changes in human supraclavicular fat.

**Table 2:** Comparison of advantages and weaknesses of existing MR methods for BAT research.

	**Differentiating BAT/WAT at rest**	**BAT activation detection**	**How quantitative at the moment**	**Availability**	**Motionrobustness**	**Temporalresolution**	**Spatialresolution**	**Specificityfor BAT**	**Other physiological confounders**	**Contrast agent-free**
**BAT Morphology**										
PDFF^ 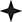 ^	−	+	++	(+)	+	−	++	−		✓
T2* mapping^ 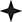 ^	+	(−)	−	(+)	+	−	++	(+)	Perfusion	✓
DW-MRS of fat^ 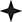 ^	++	−	+	+	−−	−	−−	+		✓
DW-MRS of water^ 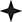 ^	+	−	−	+	−	−	−−	(+)		✓
**BAT Perfusion + Oxygenation**										
DCE^ 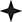 ^	+	++	−	+	+	++	+	+		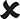
ASL^ 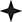 ^	+	++	−	+	−	+	+	+		✓
BOLD^ 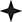 ^	−	+	−	+	−−	+	++	(+)	Microstructure	✓
Xenon^ 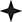 ^ (Perfusion rodents with respiratoy trigger)	+	++	++	−	++	+	−	+		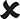
Xenon^ 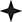 ^ (Perfusion Humans)	+	++	+	−	++	++	−−	+		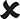
**BAT Thermometry**										
PRFS^ 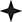 ^	−−	(+)	(−)	+	−−	−	+	(+)	Many (190)	✓
Xenon^ 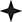 ^ (Thermometry)	−	++	++	−	++	++	−	++		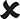
iMQC^ 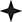 ^	+	+	(+)	(+)	++	−−	−−	(+)	Fat susceptibility	✓
**BAT Metabolism**										
^31^P	−	(−)	(+)	(+)	+	−−	−−	+	Partial volume	✓
^13^C	−	+	(+)	−−	++	++	−−	+	Fast metabolic dynamics	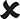
^2^H^ 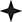 ^ (^2^H-glucose)	−	+	−	−	−	−−	−−	−	Fat peak contaminates metabolites	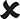

*Grades were given ranging from “−−” for very bad performance to “++” for very good performance. The parentheses indicate that the statement holds true only under certain circumstances. Techniques that the co-authors have experience with to study BAT are marked with a^
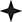
^*.

Another issue faced by current biomedical imaging research of BAT is the lack of a good animal model. Most of the techniques have been validated in mice or rats that are young, fed on a low-calorie diet, and living under constant thermal stress, i.e., under thermal and nutritional conditions that are very far from human conditions. Thus, their BAT morphology (tissue fat content, average cell size, mitochondria content, and vascularization) and thermogenic capacity (UCP1 content) are different from that of adult humans. It should therefore come as no surprise if human translations of these techniques do not turn out as expected. As animal models have been extremely valuable for developing novel MR methodologies, the choice of the appropriate animal model should be carefully considered before human translation of these technologies. For example, humanized mice, i.e., mice fed with a Western diet and kept at a thermoneutral condition, whose BAT better resembles that of adult humans, could be a better choice than chow fed mice kept at a standard laboratory temperature.

### MR Experimental Challenges

Because of its location, right above the chest cavity, BAT is highly susceptible to motion, which can lead to tissue displacement and consequent field variations during image acquisition (Supplementary Material 1). In addition, organized around major vessels, which supply the upper extremities and the head with blood, the tissue is also extremely sensitive to pulsation artifacts (Supplementary Material 2). If no triggering or other motion control is done, motion can significantly blur any tissue maps and introduce errors in the quantitative measurements. Ghosting artifacts originating from periodic respiratory motion can be reduced either by avoiding the phase encoding direction along the axis of motion (most severe in feet/head direction) or by a respiratory-triggered acquisition. The sensitivity to any motion becomes especially evident when several images are required to generate one quantitative map, including DWI, MR fat fraction maps, T_2_^*^ mapping, etc. In case of diffusion contrast, physiological motion may be misinterpreted as diffusion, causing an overestimation of the diffusion parameter. In case of phase-sensitive acquisitions such as fat fraction and T_2_^*^ mapping, the periodic field variations may hamper accurate estimation of these parameters. How respiratory motion-induced tissue displacement and main magnetic field fluctuations influence the estimated fat fraction remains a critical unanswered question and needs to be addressed in future studies (192).

## Future Perspectives

MRI offers the possibility to perform larger and longitudinal studies in healthy cohorts as it does not use ionizing radiation and, usually, does not need injections of contrast material. Longitudinal studies could help in assessing the efficacy of BAT targeting therapies, as well as understanding the role of BAT within the complex human physiology.

However, cost, long scan times, and the need to use specific sequences limit the application of MRI in large cohorts, so most MRI studies published so far are reported on smaller numbers of participants (*n* < 100).

BAT imaging is challenging because of its temporal heterogeneity: Because BAT can be regarded as a highly active organ, its presence, morphology, and activity strongly depend on a range of environmental (weather, season) and hormonal (for instance postprandial, menstrual cycle) conditions. While future imaging study designs should pay more attention to these factors, these factors may also open many doors for potential interesting investigations. Certainly, when doing activation studies, MR methods will need to improve in temporal resolution.

BAT imaging is also challenging because of its spatial heterogeneity: Because BAT is highly vascularized, and interweaved with WAT, MR methods for characterizing BAT will highly profit from a higher spatial resolution, avoiding partial volume effects. This could be realized by translation to higher magnetic fields, the use of superconducting RF receive coils (193, 194), more advanced acquisition and reconstruction techniques involving parallel imaging (195) and compressed sensing (196), and better signal modeling (197) in case of quantitative MR parameter fitting.

BAT imaging is also challenging because of motion influences from both breathing and vessel pulsation. Instead of simply translating quantitative parameter mapping methods established in examinations of other more rigid body parts, such as the brain or the musculoskeletal system, more inspiration could be found from cardiac MR.

So far, the implemented MR methods have been more reliable in detecting activated BAT vs. non-activated BAT compared to differentiating non-activated BAT from WAT (99, 102). One striking reason for this is the fact that the allegedly quantitative methods were in fact rather qualitative, providing only a contrast between the activated and the non-activated state. Good news is that the development of quantitative MR methods is not for a long time yet exhausted. There is a lot of promising work left to do in the entire pipeline from clever sequence design, over parameter modeling and image analysis.

For making MR research of BAT a success story, emphasis must be placed on the continuous development of robust and reproducible quantitative methods.

## Conclusion

MRI and MRS provide a wide range of tools for assessing various aspects of both BAT morphometry and function. They are very attractive because they have no limitation in imaging penetration depth, do not deliver any mutagenic radiation, and work with spins residing naturally in the body as well as with a range of MR-sensitive tracers. MR methods to assess BAT microstructure include fat fraction mapping, T2^*^ mapping, diffusion imaging, and iMQC imaging. BAT metabolic activity can be examined directly with ^31^P, ^2^H, and hyperpolarized ^13^C MRI and MRS, and indirectly via perfusion sensitive sequences including DCE, QSM, T2^*^, and ^129^Xe. MR thermometry was successfully used to observe thermogenesis of BAT using PRFS in rodents and ^129^Xe in humans. Despite the prosperous reports of MR studies of BAT, the wider adoption of many of these MR techniques requires further validation, including fat fraction mapping. Even though it is currently the most widely used technique, special care is required due to the here discussed limitations in quantifying the BAT fraction in the human supraclavicular fossa. Nonetheless, the versatility of possible MRI contrast mechanisms and the non-invasive character of MRI remain significant advocates for supporting continuous technical developments for MRI methods in the analysis of BAT morphometry and function.

## Author Contributions

DK devised the outline of the manuscript. All authors contributed to drafting a first version. RB and MW created the figures for the manuscript. RB, DK, and MW worked on continuous revision of the manuscript. Everyone read and approved the submitted version.

## Conflict of Interest

The authors declare that the research was conducted in the absence of any commercial or financial relationships that could be construed as a potential conflict of interest.

## Supplementary Material

Supplementary Material 1 Illustration of respiratory motion induced effects on quantitative maps in the supraclavicular region by breath hold scans at full inspiration and full expiration using a 6-echo data set. **(A)** PDFF maps of the same slice show anatomical variations comparing the scan from inspiration and expiration state. **(B)** The corresponding calculated field maps from the different respiration states show a large field variation within one image due to the complex geometry in that region including the proximity to the lungs. **(C)** The large difference of the field maps between inspiration and expiration indicates large respiratory-induced field fluctuations that may cause quantification errors when not accounted for.

Supplementary Material 2 The video shows the pulsation effect of the large vessels within the supraclavicular fossa causing severe tissue deformation in the neighboring tissue. This is challenging in the context of diffusion-weighted acquisitions and quite similar to the motion problem faced during cardiac imaging.

## Abbreviations

ADC: apparent diffusion coefficient
ASL: arterial spin labeling
BAT: brown adipose tissue
BOLD: blood oxygenation level dependent
CEUS: contrast-enhanced ultrasound
CLI: Cerenkov luminescence imaging
CT: computed tomography
DCE-MRI: dynamic contrast-enhanced magnetic resonance imaging
DECT: dual-energy CT
DMI: deuterium metabolic imaging
DWI: diffusion-weighted imaging
DW-MRS: diffusion-weighted magnetic resonance spectroscopy
^18^F-FDG: fluorine-18 fluorodeoxyglucose
fMRI: functional magnetic resonance imaging
HP: hyperpolarized
HU: Hounsfield units
iBAT: interscapular BAT
iMQC: intermolecular multiple quantum coherence
iZQC: intermolecular zero quantum coherence
MION: monocrystalline iron oxide nanoparticles
MRI: magnetic resonance imaging
MRS: magnetic resonance spectroscopy
MSOT: multispectral optoacoustic tomography
NIR_TRS_: near-infrared time-resolved spectroscopy
PDFF: proton density fat fraction
PET: positron emission tomography
PRFS: proton resonance frequency shift
QSM: quantitative susceptibility mapping
SNR: signal-to-noise ratio
SPION: superparamagnetic iron oxide nanoparticles
SUV: standardized uptake values
TE: echo time
TR: repetition time
TRL-^59^FE-SPIO: triglyceride-rich lipoprotein-^59^FE-SPIOs
UCP-1: uncoupling protein-1
WAT: white adipose tissue
ZSI: Z-spectrum imaging.
