# Supplementary figures and images for: Magnetic Resonance Imaging Techniques for Brown Adipose Tissue Detection
Source: Front Endocrinol (Lausanne). 2020 Aug 7;11:421. doi: 10.3389/fendo.2020.00421 (PMC7426399; doi:10.3389/fendo.2020.00421)

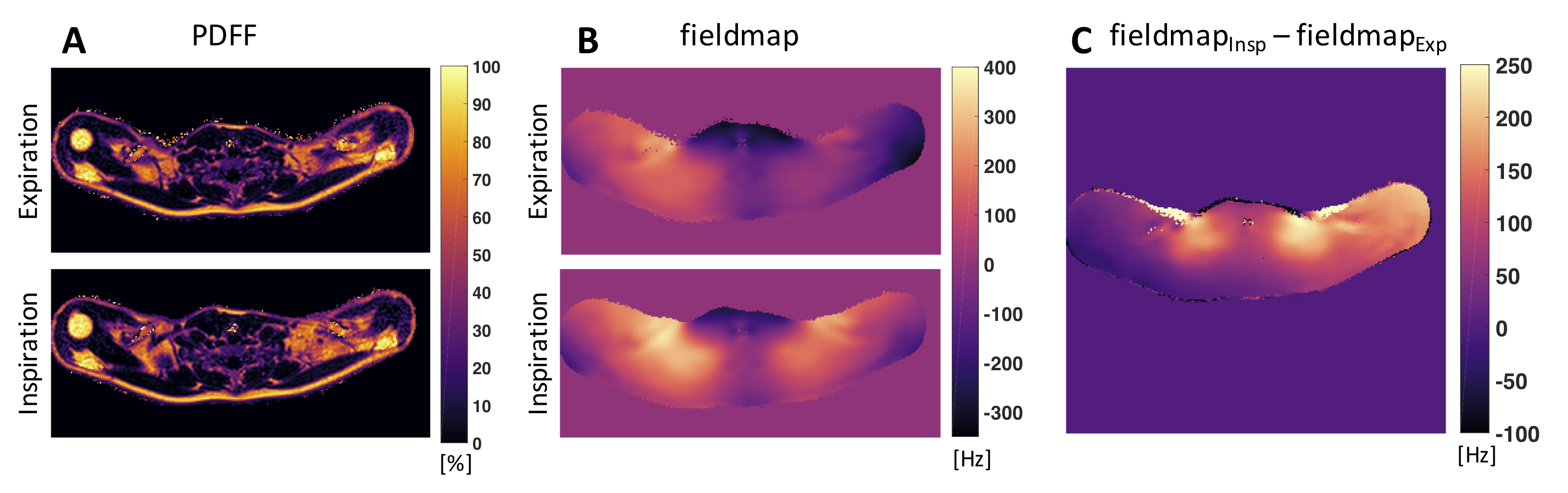

Supplement: Supplementary Material 1 — Illustration of respiratory motion induced effects on quantitative maps in the supraclavicular region by breath hold scans at full inspiration and full expiration using a 6-echo data set. (A) PDFF maps of the same slice show anatomical variations comparing the scan from inspiration and expiration state. (B) The corresponding calculated field maps from the different respiration states show a large field variation within one image due to the complex geometry in that region including the proximity to the lungs. (C) The large difference of the field maps between inspiration and expiration indicates large respiratory-induced field fluctuations that may cause quantification errors when not accounted for. [file Image_1.TIFF]
